# Developing a short-form version of the HIV Disability Questionnaire (SF-HDQ) for use in clinical practice: a Rasch analysis

**DOI:** 10.1186/s12955-020-01643-2

**Published:** 2021-01-06

**Authors:** Kelly K. O’Brien, Mendwas Dzingina, Richard Harding, Wei Gao, Eve Namisango, Lisa Avery, Aileen M. Davis

**Affiliations:** 1 Department of Physical Therapy, University of Toronto, 160-500 University Avenue, Toronto, ON M5G 1V7 Canada; 2 Institute of Health Policy, Management and Evaluation (IHPME), University of Toronto, Toronto, ON Canada; 3 Rehabilitation Sciences Institute (RSI), University of Toronto, Toronto, ON Canada; 4 Florence Nightingale Faculty of Nursing Midwifery and Palliative Care, Cicely Saunders Institute, King’s College London, London, UK; 5 Avery Information, Toronto, ON Canada; 6 Health Care and Outcomes Research, Krembil Research Institute, University Health Network, Toronto, ON Canada

**Keywords:** Disability evaluation, Outcome measures, Patient reported outcomes, Item analysis, Item response theory

## Abstract

**Background:**

Disability is an increasingly important health-related outcome to consider as more individuals are now aging with Human Immunodeficiency Virus (HIV) and multimorbidity. The HIV Disability Questionnaire (HDQ) is a patient-reported outcome measure (PROM), developed to measure the presence, severity and episodic nature of disability among adults living with HIV. The 69-item HDQ includes six domains: physical, cognitive, mental-emotional symptoms and impairments, uncertainty and worrying about the future, difficulties with day-to-day activities, and challenges to social inclusion. Our aim was to develop a short-form version of the HIV Disability Questionnaire (SF-HDQ) to facilitate use in clinical and community-based practice among adults living with HIV.

**Methods:**

We used Rasch analysis to inform item reduction using an existing dataset of adults living with HIV in Canada (n = 941) and Ireland (n = 96) who completed the HDQ (n = 1037). We evaluated overall model fit with Cronbach’s alpha and Person Separation Indices (PSIs) (≥ 0.70 acceptable). Individual items were evaluated for item threshold ordering, fit residuals, differential item functioning (DIF) and unidimensionality. For item threshold ordering, we examined item characteristic curves and threshold maps merging response options of items with disordered thresholds to obtain order. Items with fit residuals > 2.5 or less than − 2.5 and statistically significant after Bonferroni-adjustment were considered for removal. For DIF, we considered removing items with response patterns that varied according to country, age group (≥ 50 years versus < 50 years), and gender. Subscales were considered unidimensional if ≤ 5% of t-tests comparing possible patterns in residuals were significant.

**Results:**

We removed 34 items, resulting in a 35-item SF-HDQ with domain structure: physical (10 items); cognitive (3 items); mental-emotional (5 items); uncertainty (5 items); difficulties with day-to-day activities (5 items) and challenges to social inclusion (7 items). Overall models’ fit: Cronbach’s alphas ranged from 0.78 (cognitive) to 0.85 (physical and mental-emotional) and PSIs from 0.69 (day-to-day activities) to 0.79 (physical and mental-emotional). Three items were rescored to achieve ordered thresholds. All domains demonstrated unidimensionality. Three items with DIF were retained because of their clinical importance.

**Conclusion:**

The 35-item SF-HDQ offers a brief, comprehensive disability PROM for use in clinical and community-based practice with adults living with HIV.

## Background

In countries with access to antiretroviral therapy, HIV is now experienced as a chronic illness with more adults aging with Human Immunodeficiency Virus (HIV) [1–4]. Chronic conditions are more prevalent among people aging with HIV than the general population [5, 6] such as cardiovascular disease [7], bone and joint disorders [8, 9], diabetes [10], frailty [11], neurocognitive disorders [12, 13], and some forms of cancer [14]. This multimorbidity can increase the complexities of aging with HIV [15–18], collectively referred to as disability [17, 19]. Adults aging with HIV can face additional challenges of ageism, stigma, mental health issues, financial insecurity, access to long-term care housing, and lack of social support, adding further to the complexity of disability over the aging life course [20–23]. Hence, the emerging needs of adults aging with HIV require personalized and preventative approaches to multi-morbidity and disability.

The *Episodic Disability Framework* is a conceptual framework developed from the perspective of adults living with HIV that characterizes the multidimensional and episodic nature of disability [17, 19]. Dimensions of disability in the Framework include physical, cognitive, mental and emotional symptoms and impairments, difficulties carrying out day-to-day activities, challenges to social inclusion, and uncertainty about future health [17, 19]. This Framework, considered novel for its inclusion of uncertainty a key dimension of aging with chronic illness, conceptually underpinned the development of a new HIV-disability patient-reported outcome measure (PROM) [24].

Using categories in the *Episodic Disability Framework*, members of our team established the HIV Disability Questionnaire (HDQ), a PROM developed to describe the presence, severity and episodic nature of disability experienced living with HIV [24]. As the only known HIV-specific disability PROM derived and validated from the perspectives of adults living with HIV [24], the HDQ addresses gaps in previously existing health status measures by capturing uncertainty about the future and challenges to social inclusion [25]. The HDQ possesses sensibility, reliability and validity for use among community dwelling adults living with HIV in Canada, Ireland, the United States and United Kingdom, suggesting its international applicability and scope [26–29]. While the HDQ has potential for clinical utility, concerns exist that it is too lengthy and not feasible for use in the busy clinic or community-based setting [30]. Our aim was to develop a Short-Form version of the HIV Disability Questionnaire (SF-HDQ) to facilitate use in clinical and community-based practice among adults living with HIV.

## Methods

We conducted a secondary analysis using data from adults living with HIV in Canada (n = 941) and Ireland (n = 96) who completed the HDQ, and fitted the data to the Rasch model to develop the SF-HDQ [31].

*HIV Disability Questionnaire* The HDQ includes 69-items grouped into six domains: (1) physical symptoms and impairments (20 items), (2) cognitive symptoms and impairments (3 items), (3) mental-emotional symptoms and impairments (11 items), (4) uncertainty about future health (14 items), (5) difficulties with day-to-day activities (9 items), and (6) challenges to social inclusion (12 items) [25, 32]. The questionnaire describes a range of health challenges a person might experience so that clinicians may better understand and address the disability needs of people aging with HIV. The HDQ possesses a 5-point ordinal response scale that measures the presence and severity of disability ranging from “0 = not at all” to “4 = an extreme amount”, and a binary response scale that captures the episodic nature of disability (yes = 1 or no = 0). The HDQ is scored as a simple sum transformed out of 100 whereby higher scores (range 0–100) indicate a higher presence, severity and episodic nature of disability.

### Rasch analysis

We conducted a Rasch analysis, a popular method for developing PROMs, assessing cross-cultural validity, and guiding item reduction [31, 33–36]. Our approach to the conduct and reporting of this Rasch analysis were informed by the work and guidelines for reporting studies using Rasch analysis, developed by Alan Tennant [31, 33, 34, 37, 38]. The Rasch model uses a logistic function to indicate the probability that an individual responds to a particular item (response option) and is dependent on both the *individual ability* and the *difficulty (or severity)* of the item [39]. Items representing the latent construct (disability) are hierarchically ordered along the continuum of difficulty. Fitting ordinal response data, such as items in the HDQ, to the Rasch model transforms the score into an interval scale. As the items are hierarchically ordered, Rasch analysis allows an individual’s location on the continuum to be precisely estimated using few items, making it an ideal approach for item reduction [33–36]. For the development of the SF-HDQ, we conducted a Rasch analysis using a rating scale model [40] focused on the severity scale (0–4 response options). Given the Rasch model assumes unidimensionality, and our assumption that each domain of disability measures a distinct construct of disability, we conducted our analysis on each domain separately.

For each HDQ domain, we conducted the following analytical steps iteratively to examine overall model and item fit:

1. *Rasch model fit* We evaluated model fit statistics including Cronbach’s alpha and Person Separation Indices (PSI) to determine the extent to which the domains and individual items fit the Rasch model. We considered ≥ 0.70 as acceptable [31, 41]. We assessed the Chi Square statistic (χ^2^) with Bonferroni adjusted significance levels (alpha value = 0.05/number of items in the original domain), but did not consider it a determinant of model fit given its sensitivity to large sample sizes, which can overestimate lack of model fit [42].
2. *Item thresholds* We identified items with disordered thresholds, which suggest respondents may not be able to differentiate between response options (or degrees of disability severity). In such instances, if clinically meaningful, we merged response options of items with disordered thresholds to obtain ordered thresholds. Reordered items that still did not achieve ordered thresholds were considered for deletion [41].
  We also examined the difficulty hierarchy of items by examining item characteristics curves and threshold maps illustrating the spread of logit values of all items for each domain.
3. *Item fit statistics* We examined observed and expected values and considered items with absolute standardized fit residuals > 2.5 for deletion [43, 44].
4. *Differential item functioning (DIF)* We examined measurement invariance and considered removing items with response patterns that varied according to country (Canada versus Ireland), age group (≥ 50 years versus < 50 years), and gender [34]. Items with significant DIF and > 1.0 logit difference between groups were considered for deletion [34].
5. *Unidimensionality* For each domain, we conducted a principal components analysis (PCA) of the residuals to ensure there were no additional latent factors after the Rasch factor had been accounted for in the model. We identified two item sets from the PCA (positively and negatively correlated items with the residuals) to derive separate person estimates and then conducted independent t-tests to determine the number of cases that significantly differed (0.05 level). Strict unidimensionality was confirmed when ≤ 5% of independent t-tests comparing possible patterns in residuals were significant [31, 37, 45].

We used RUMM2030 for the analysis [46]. We used a combination of Rasch and clinical reasoning to inform our final decisions to remove or retain items. We removed items considered for deletion one at a time and re-evaluated model and item-level fit after each item removal in order to identify the ideal domain item composition. We did not remove any items from the cognitive domain as we considered three items as the minimum number to comprise a latent variable representing a domain of disability [47, 48]. Co-authors (KKO, MD, RH, EN, GW, AMD) met on three occasions to review the Rasch model results for each domain and discussed decisions for item deletion or retention. After three iterations of preliminary model results, two authors (KKO and AMD) met to review model and item fit characteristics for each domain and to determine final item retention (or deletion), maximizing model fit while ensuring clinical relevance and utility of the questionnaire. We defined ‘clinical importance’ of an item as an item that represents a key component of the SF-HDQ domain determined by clinical and research expertise of the team, and supported by research evidence. In areas where we retained an item due to clinical importance, we provide references from the literature in the discussion to support our decision.

*Sample size* Our analysis was based on the assumption that at least 10 observations per response option are needed for item threshold analysis [49] and at least 50 participants are needed to determine item fit with the Rasch model [50]. Hence our sample of 1037, was sufficient for the analysis.

### SF-HDQ scoring

We created a user-friendly scoring algorithm for each domain to convert raw summed SF-HDQ scores to the equivalent Rasch-based person logit scores. Using methodology described by Perruccio et al. [51], we fit a cubic function to regress Rasch-based person logit scores on the raw summed SF-HDQ scores, which was then transformed to an interval scale range of 0–100. The resulting formula for each SF-HDQ domain can then be used to yield simple Rasch-based interval SF-HDQ domain scores (range: 0–100) with higher scores indicating greater disability.

## Results

### Sample characteristics

The majority of participants were men (811/1037; 78%), median age of 47 years, of which 41% (427/1307) were ≥ 50 years of age. Participants reported living with a median of three concurrent health conditions in addition to HIV; most common conditions included mental health (e.g. anxiety and depression), muscle pain and joint pain. Description of participant characteristics based on country are provided in Table 1. Further detail on the characteristics of these sample populations have been published elsewhere [26, 52].

**Table 1:** Characteristics of participants by country

Characteristic	Canada (n = 941)	Ireland (n = 96)
	Number (%)	Number (%)
Gender		
Men	740 (79%)	71 (74%)
Women	159 (17%)	23 (24%)
Transgender	19 (2%)	2 (2%)
Two-spirited	15 (2%)	–
Missing	8 (1%)	–
Median age in years (25–75th percentile)	48 (39–54)	41 (34–48)
50 years or older	405 (43%)	22 (23%)
Median time since HIV diagnosis in years (25–75th percentile)	13 (6–21)	9 (4–14)
Taking antiretroviral therapy	851 (90%)	84 (88%)
Undetectable viral load (< 40 copies/mL)^b^	572 (67%)	41 (85%)
Employment status (full-time or part-time)	350 (37%)	52 (54%)
Median number of concurrent health conditions (25–75th percentile)	3 (1–6)	1 (0–3)
Living with ≥ 2 concurrent health conditions in addition to HIV	518 (72%)	39 (41%)
Common concurrent health conditions^a^		
Mental health condition	392 (42%)	18 (19%)
Muscle pain	308 (33%)	21 (22%)
Joint pain	282 (30%)	22 (23%)
Addiction	248 (26%)	9 (9%)
Neurocognitive decline	209 (22%)	11 (12%)
Hepatitis C^c^	95 (10%)	21 (22%)
Self-rated health status ‘very good’	274 (29%)	34 (35%)

Number of participants: n = 1037; Not all characteristics add to the total n due to missing responses

^a^Concurrent health conditions experienced by ≥ 20% of respondents in either sample

^b^Based on number of participants who confirmed they were taking antiretroviral therapy; out of 851 (Canadian) and 48 (Irish) samples respectively. Among Canadian sample, 44 (5%) could not remember or did not know their viral load

^c^Irish sample recruited from an HIV-Hep-C co-infection clinic

### Rasch models for SF-HDQ domains

Overall across all six domains, we removed 34 items, resulting in a 35-item SF-HDQ with domain structure: physical (20 items reduced to 10); cognitive (3 items; none removed); mental-emotional (11 items reduced to 5); uncertainty (14 items reduced to 5); difficulties with day-to-day activities (9 items reduced to 5) and challenges to social inclusion (12 items reduced to 7).

**Fig. 1:**
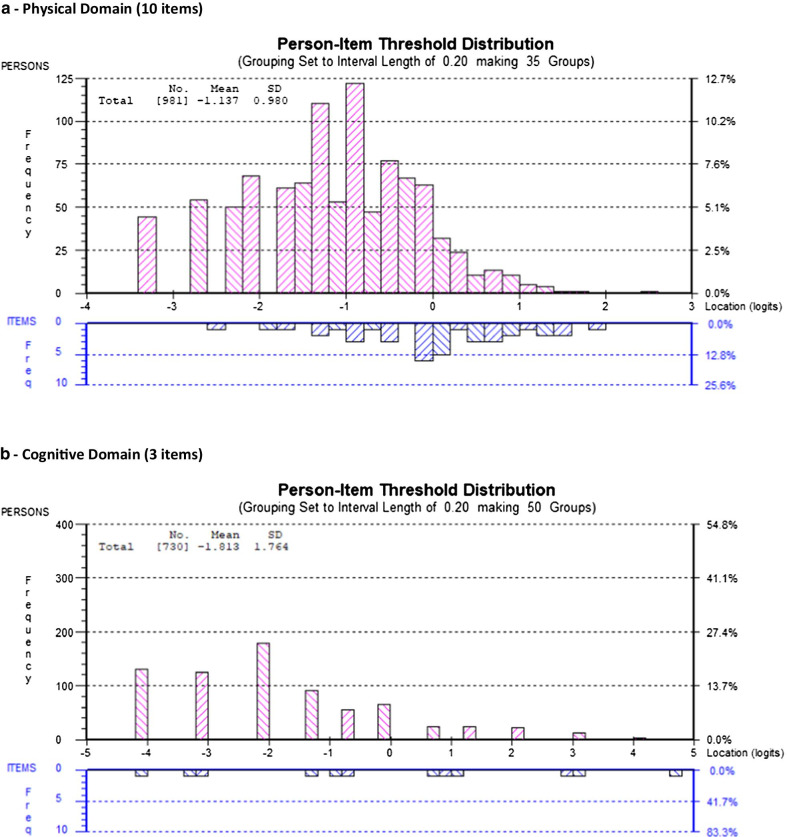

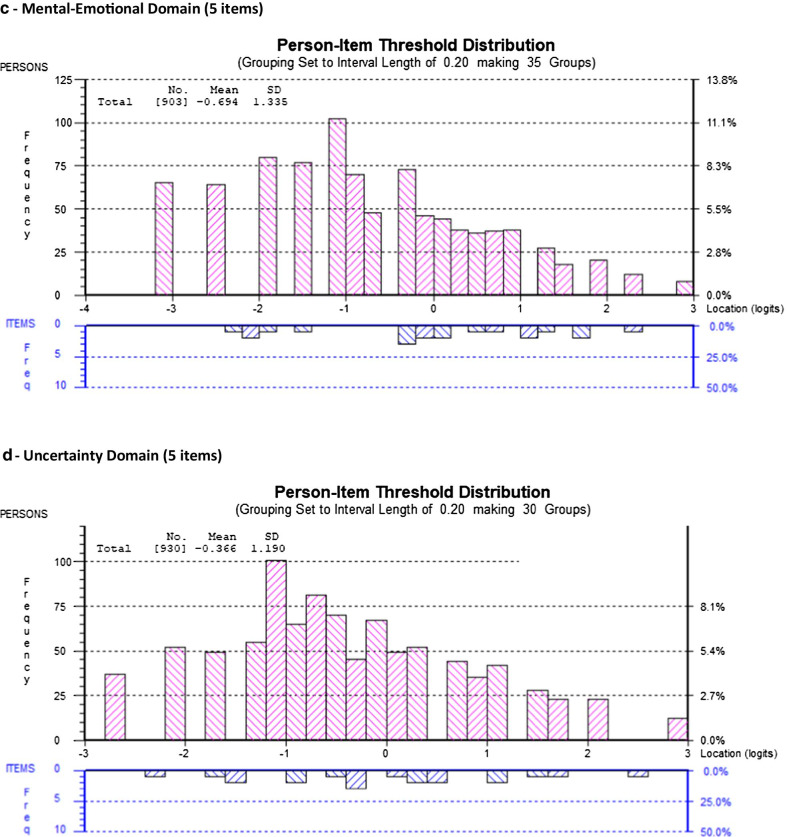

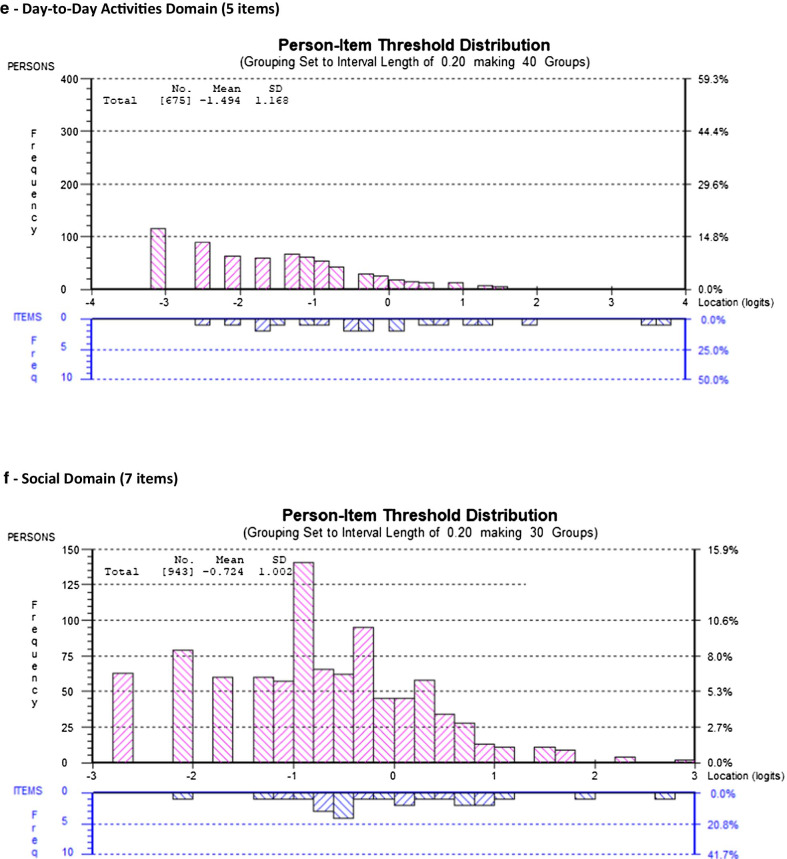
Person-item threshold distributions for final SF-HDQ domains

For all six models, Fig. 1 includes the person-item threshold distribution for each domain in the final models showing the distribution of item-levels (easiest to most difficult) for participants in the sample (Fig. 1a–f).

**Fig. 2:**
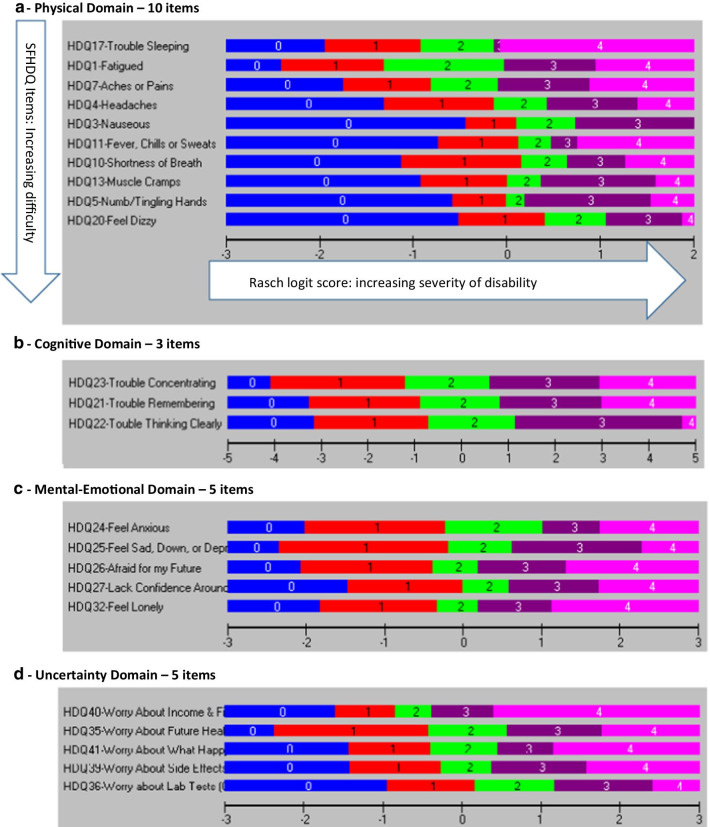

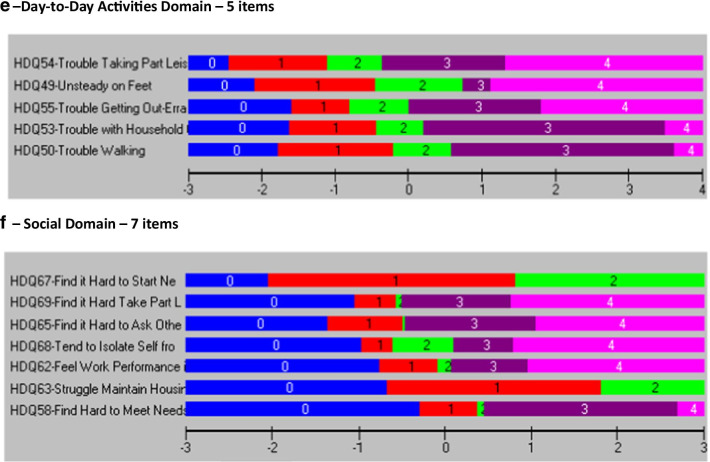
Item threshold map of the final model SF-HDQ domains in order of item difficulty. This figure illustrates the item threshold map for each final SF-HDQ domain in order of increasing difficulty from top to bottom, and with severity levels increasing from left to right (**a**–**f**). For example, in the physical domain 'trouble sleeping' is the item with least difficulty to 'feeling dizzy' which is the item with the most difficulty to score high levels of disability severity

Figure 2 illustrates the item threshold map for each final SF-HDQ domain in order of increasing difficulty from top to bottom, and with severity levels increasing from left to right (Fig. 2a–f).

Additional file 1 presents the model characteristics and fit statistics from the first to final iteration of the Rasch model for each domain outlining the step-wise process and decision-making to remove items. Additional file 2 includes the category probability curves for each item in the final SF-HDQ models. Additional file 3 provides an overview of the original HDQ items (69 items) and final proposed SF-HDQ items (35 items).

Tables 2, 3, 4, 5, 6 and 7 provides an overview of model and item fit statistics for the resulting SF-HDQ items in each domain. Below we describe the Rasch model results for each domain.

#### Physical domain (20 items to 10 items)

In the initial model (20 items) (Cronbach’s alpha: 0.903; PSI: 0.869), three of the 20 items in the physical domain (HDQ3-nausea, HDQ8-trouble swallowing, and HDQ15 – unintentionally losing weight) were disordered (Additional file 1; Physical Domain Initial Model 1). Upon examination of the item category probability curves (not shown), we reordered HDQ3 (nausea) by collapsing response category ‘3’ and ‘4’ (4 response categories); and HDQ8 (trouble swallowing) by collapsing response categories 1, 2, and 3 (3 response categories). HDQ15 (intentionally losing weight) did not make sense with reordering, hence we removed this item.

Among the remaining 19 items, we deleted 7 items in a step-wise fashion with fit residuals >  ± 2.5: HDQ9 (decreased libido); HDQ2 (diarrhea); HDQ12 (weakness in muscles); HDQ8 (trouble swallowing); HDQ18 (vision problems); HDQ19 (trouble hearing); and HDQ16 (lack of appetite) (12-item model). We deleted HDQ6 (numbness or tingling in feet) due to DIF (age group) and removed HDQ6 (numbness or tingling in my feet) in order to merge with HDQ5 (numbness or tingling in hands) which we will refine to one SF-HDQ item (numbness or tingling in my hands or feet) in a future iteration of the tool. We deleted HDQ14 (stomach cramps) due to DIF (age group) and retained HDQ13 (muscle cramps) despite having a large residual due to its clinical importance and lack of strong correlation with HDQ7 (aches and pains) (not shown) suggesting muscle cramps is a distinct concept. We did not merge muscle and stomach cramp items given these items refer to distinct sources of pain. The final physical domain included 10 items. The 10-item model achieved adequate model and item fit statistics, unidimensionality, and no items with DIF (Table 2; Figs. 1a, 2a; Additional files 1, 2).

**Table 2:** Physical Domain (10 items) - Overview of final SF-HDQ item level and domain model fit statistics

Item #	Final SF-HDQ items	Item level statistics				
Physical domain		Location	SE	Residual	ChiSq χ^2^	*p* value
1	Fatigue	− 0.694	0.038	− 0.276	13.544	0.140
3	Nausea^a^	0.132	0.044	− 0.748	18.549	0.029
4	Headaches	0.098	0.039	0.361	16.422	0.059
5	Numbness or tingling in hands	0.288	0.039	0.100	8.470	0.488
7	Aches or pains	− 0.441	0.036	− 1.399	21.204	0.012
10	Shortness of breath	0.240	0.041	0.061	15.153	0.087
11	Fever, chills, or sweats	0.162	0.039	− 0.529	18.191	0.033
13	Muscle cramps	0.262	0.040	− 2.120	13.636	0.136
17	Trouble sleeping	− 0.761	0.034	2.141	14.346	0.111
20	Feel dizzy	0.714	0.045	− 1.555	14.747	0.098
Final model: physical domain—10 items (raw score range: 0–39)						
Mean		− 1.137				
Standard deviation		0.980				
Sample size		981				
Chi-square statistic (*df*) *p* value		154.2609 (*df*: 90) *p* = 0.0003				
Cronbach’s alpha		0.84762				
Person Separation Index		0.79042				
Unidimensionality t-test (% significant)		0.41%				
Differential Item Functioning (DIF)		None				

^a^HDQ3 rescored to 4 response categories (0–3)

#### Cognitive domain (3 items)

We did not remove any items from the cognitive domain as the original scale included 3 items. The 3-item model achieved adequate fit statistics and no items with DIF. One item (HDQ21-trouble remembering) had a standardized fit residual > 2.5, however we retained this item due to clinical importance and the requirement for the minimum of 3 items required to comprise this domain (Table 3; Figs. 1b, 2b; Additional files 1, 2).

**Table 3:** Cognitive Domain (3 items) - Overview of final SF-HDQ item level and domain model fit statistics

Item #	Final SF-HDQ items	Item level statistics				
Cognitive domain		Location	SE	Residual	ChiSq χ ^2^	*p* value
21	Trouble remembering like appointments and when to take medication	− 0.072	0.058	2.764*	20.271	0.005*
22	Trouble thinking clearly	0.498	0.061	− 1.078	34.33	< 0.001^a^
23	Trouble concentrating	− 0.427	0.060	− 0.918	21.292	0.003^a^
Final model: cognitive domain—3 items (raw score range: 0–12)						
Mean		− 1.813				
Standard deviation		1.764				
Sample size		730				
Chi-square statistic (*df*) *p* value		75.8970 (*df*: 21); *p* < 0.001				
Cronbach’s alpha		0.77602				
Person Separation Index		0.70994				
Unidimensionality t-test (% significant)		1.23%				
Differential Item Functioning (DIF)		None				

^*^Item fit residual for HDQ21 (2.764) significant (p < 0.017; Bonferroni: 0.05/3) but retained due to clinical importance and need for minimum of 3 items in domain

^a^Significant residual (p < 0.017; Bonferroni: 0.05/3) but fit residual *F* value <  ± 2.5

#### Mental-emotional domain (11 items to 5 items)

In the initial model (11 items) (Cronbach alpha: 0.928; PSI: 0.891), no items in the mental-emotional domain were disordered; there were 5 items with fit residuals >  ± 2.5 statistically significant after Bonferroni adjustment (Additional file 1; Mental-Emotional Domain Initial Model 1). Among the original 11 items, we deleted 4 items in a step-wise fashion with fit residuals >  ± 2.5 and/or statistically significant after Bonferroni-adjustment (in order from greatest residuals): HDQ28 (uncomfortable with how my body looks); HDQ33 (discouraged about future life options); HDQ29 (feel isolated even when around others); HDQ34 (feel shut out by friends or family); and HDQ30 (feel embarrassed around others) resulting in 6 remaining items. We subsequently deleted HDQ31 (feel guilty) as it had a high item fit residual (3.02) even though it was not significant, due to its lack of clinical importance in relation to other items. The final mental-emotional domain included 5 items. The 5-item model achieved adequate fit statistics and no items with DIF (Table 4; Figs. 1c, 2c; Additional files 1, 2).

**Table 4:** Mental-Emotional Domain (5 items) - Overview of final SF-HDQ item level and domain model fit statistics

Item #	Final SF-HDQ items	Item level statistics				
Mental-emotional domain		Location	SE	Residual	ChiSq χ ^2^	*p* value
24	Feel anxious	0.128	0.044	0.385	11.297	0.256
25	Feel sad, down, or depressed	0.092	0.044	− 1.798	20.480	0.015
26	Afraid for my future	− 0.232	0.041	0.088	5.906	0.749
27	Lack confidence around others	0.216	0.042	0.610	9.372	0.404
32	Feel lonely	− 0.203	0.04	1.256	6.858	0.652
Final model: mental-emotional domain—5 items (raw score range: 0–20)						
Mean		-0.694				
Standard deviation		1.335				
Sample size		903				
Chi-square statistic (*df*) *p* value		53.9124 (*df*: 45) *p* = 0.170297^a^				
Cronbach’s alpha		0.84935				
Person Separation Index		0.79154				
Unidimensionality t-test (% significant)		1.14%				
Differential Item Functioning (DIF)		None				

^a^Chi-square statistic not significant (ideal outcome)

#### Uncertainty (14 items to 5 items)

In the initial model (14 items) (Cronbach’s alpha: 0.918; PSI: 0.899), 5 items were disordered (HDQ42—worry about remaining in the workforce, volunteering or school; HDQ43-worry about dying; HDQ45—worry about legal issues related to HIV disclosure; HDQ47—worry about transmitting HIV; and HDQ48-putting life decisions on hold) (Additional file 1; Uncertainty Domain Initial Model 1). Upon examination of the item category probability curves (not shown), an attempt to reorder categories in items HDQ42, HDQ47 and HDQ48 was non sensical, hence we removed these items. Among the remaining 11 items, we rescored HDQ43 and HDQ45 by collapsing response options ‘3’ and ‘4’ (resulting in 4 response options). We subsequently deleted 5 items in a stepwise fashion with fit residuals >  ± 2.5 and/or with significance after Bonferroni adjustment (in order from greatest residuals): HDQ46 (worry about what others think if they knew I was HIV positive); HDQ44 (worry about bodily appearance); HDQ45 (worry about legal issues of telling others about HIV status); HDQ37 (worry about having a serious illness); and HDQ38 (worry about what the outcome of my next episodic of illness might be).

Among the remaining 6 items, we deleted HDQ43 (worry about dying) as conceptually we considered this item could be captured in HDQ35 (worry about my future health). The final uncertainty domain included 5 items. The 5-item model achieved adequate fit statistics and no significant DIF (Table 5; Figs. 1d, 2d; Additional files 1, 2).

**Table 5:** Uncertainty Domain (5 items) - Overview of final SF-HDQ item level and domain model fit statistics

Item #	Final SF-HDQ items	Item level statistics				
Uncertainty domain		Location	SE	Residual	ChiSq χ^2^	*p* value
35	Worry about future health living with HIV	− 0.114	0.041	− 1.295	25.455	0.002^a^
36	Worry about lab test results such as my CD4 count and viral load	0.701	0.041	0.084	13.146	0.156
39	Worry about the side effects of HIV treatments	0.069	0.038	0.793	5.110	0.825
40	Worry about income or financial security living with HIV	− 0.608	0.035	0.900	6.796	0.658
41	Worry what might happen to my family and friends if have an episode of illness	− 0.048	0.037	0.618	7.220	0.614
Final model: uncertainty domain—5 items (raw score range: 0–20)						
Mean		− 0.366				
Standard deviation		1.190				
Sample size		930				
Chi-square statistic (*df*) *p* value		58.7265 (*df*: 45) *p* = 0.0965^b^				
Cronbach’s alpha		0.82314				
Person Separation Index		0.78006				
Unidimensionality t-test (% significant)		1.72%				
Differential Item Functioning (DIF)		None				

^a^Significant residual (*p* < 0.004; Bonferroni: 0.05/14) but fit residual *F* value <  ± 2.5

^b^Chi-square statistic not significant (ideal outcome). HDQ43—removed as this item considered to be captured by HDQ35 worry about future health

#### Difficulties with day-to-day activities (9 items to 5 items)

In the initial model (9 items) (Cronbach’s alpha: 0.881; PSI: 0.796), no items were disordered (Additional file 1; Difficulties with Day-to-Day Activities Domain Initial Model 1). Among the original 9 items, we deleted 2 items in a step-wise fashion with fit residuals >  ± 2.5 and/or with significance after Bonferroni adjustment: HDQ56 (trouble keeping track of finances); HDQ52 (trouble eating, bathing, grooming, dressing).

Among the remaining 7 items, we deleted HDQ51 (trouble climbing stairs) because of DIF for age group, and HDQ57 (trouble getting around such as driving, or taking public transport) due to DIF for country, and we considered this item conceptually as capturing part of HDQ55 (trouble getting out to do errands) as running errands involves community mobility. The final day domain included 5 items. The 5-item model achieved adequate fit statistics and no items with DIF. (Table 6; Figs. 1e, 2e; Additional files 1, 2).

**Table 6:** Day-to-Day Activities Domain (5 items) - Overview of final SF-HDQ item level and domain model fit statistics

Item #	Final SF-HDQ items	Item level statistics				
Day-to-day activities domain		Location	SE	Residual	ChiSq χ^2^	*p* value
49	Unsteady on my feet	− 0.170	0.052	0.837	8.166	0.518
50	Trouble walking	0.554	0.055	1.055	14.058	0.120
53	Trouble doing household chores such as cleaning, doing dishes, laundry, and cooking	0.407	0.052	− 1.374	27.246	0.001^a^
54	Trouble taking part in leisure or recreation, such as exercise or dancing	− 0.647	0.048	− 0.982	14.090	0.119
55	Trouble getting out to do errands, such as grocery shopping, banking, or doctor’s appointments	− 0.145	0.049	− 0.069	13.207	0.153
Final model: day-to-day activities domain—5 items (raw score range: 0–20)						
Mean		− 1.494				
Standard deviation		1.168				
Sample size		675				
Chi-square statistic (*df*) *p* value		76.7660 (*df*: 45) *p* = 0.002				
Cronbach’s alpha		0.79477				
Person Separation Index		0.69006				
Unidimensionality t-test (% significant)		0.74%				
Differential Item Functioning (DIF)		None				

Person Separatoin Index (PSI) approaching the threshold of ≥ 0.70

^a^Significant residual (*p* < 0.006; Bonferroni: 0.05/9) but fit residual *F* value <  ± 2.5

#### Social inclusion (12 item to 7 items)

In the initial model (12 items) (Cronbach’s alpha: 0.903; PSI: 0.862), 2 items were disordered (HDQ63—struggle to maintain housing; HDQ67-find it hard to start new intimate, sexual relationships living with HIV) (Additional file 1; Social Domain Initial Model 1). Upon examination of the item category probability curves (not shown), we rescored HDQ63 (housing) and HDQ67 (relationships) by collapsing response categories ‘1’ ‘2’ and ‘3’ resulting in 3 response categories for these two items.

Among the 12 items, we deleted 4 items in a step-wise fashion with fit residuals >  ± 2.5 and/or with significance after Bonferroni adjustment: HDQ64 (find it hard to talk to others about illness); HDQ59 (find hard to fulfill role as family or community member); HDQ66 (find hard to start new friendships); HDQ60 (feel cut off from friends, networks, communities).

Among the remaining 8 items, we deleted HDQ61 (illness prevents me from doing volunteer or paid work or going to school) due to DIF for country and because this item was conceptually captured by HDQ62 (work performance is limited because of my illness) and is preferred because it describes the limitation rather than absolute inability or prevention of work. The final social domain included 7 items. The 7-item model achieved adequate fit statistics. HDQ68 (tend to isolate self from others) had a significant large item fit residual after Bonferroni adjustment (F value: − 3.1; *p* = 0.003), and significant DIF for country, however if deleted from the model, the fit worsened, and we retained due to item clinical importance (Table 7; Figs. 1f, 2f; Additional files 1, 2).

**Table 7:** Social Inclusion Domain (7 items) - Overview of final SF-HDQ item level and domain model fit statistics

Item #	Final SF-HDQ items	Item level statistics				
Social domain		Location	SE	Residual	ChiSq χ^2^	*p* value
58	Find hard to meet the needs of those I care for	0.808	0.040	− 0.302	11.066	0.271
62	Feel work performance limited (0 = not at all or not applicable)	0.046	0.035	0.455	5.823	0.757
63	Struggle to maintain safe and stable housing - DIF(country)^a^	0.570	0.060	− 0.297	22.579	0.007
65	Find it hard to ask others for help when go through an episode of illness - DIF(country)	− 0.310	0.034	− 0.442	7.370	0.599
67	Find it hard to start new intimate, sexual relationships living with HIV (0 = not at all or not applicable)^a^	− 0.609	0.058	2.426	8.228	0.511
68	Tend to isolate myself from others because I am HIV positive	− 0.172	0.034	− 3.142	24.214	0.003*
69	Find it hard to take part in leisure or recreational things because can’t afford it - DIF(country)	− 0.333	0.033	0.134	6.398	0.700
Final model: social domain—7 items (raw score range: 0–24)						
Mean		− 0.724				
Standard deviation		1.002				
Sample size		943				
Chi-square statistic (*df*) *p* value		86.6772 *df*: 54; *p* = 0.025679				
Cronbach’s alpha		0.79355				
Person Separation Index		0.74478				
Unidimensionality t-test (% significant)		1.27%				
Differential Item Functioniong (DIF)		Significant DIF for country (defined as F value significant and > 1.0 logit difference between groups)—3 items (HDQ63; HDQ65; HDQ69)^b^				

^*^Residuals: HDQ68 (isolate self)—significant residual > -2.5 (*p* < 0.004; Bonferroni: 0.05/11). This item may be referring to a ‘strategy’ (isolating self) that may lead to challenges to social inclusion rather than social inclusion itself. However we retained due to clinical importance, and because if we remove this item from the model, it worsens fit (PSI = 0.676; model not shown)

Rescoring: ^a^Rescored to 3 response categories (0–2)—HDQ63 and HDQ67 to achieve ordered thresholds

HDQ61: deleted because captured in HDQ62—which describes limitations rather than prevention (all or nothing)

^b^Differential Item Functioning (DIF): Significant DIF for HDQ63, HDQ65, and HDQ69. Items retained given clinical importance and expected cultural differences related to disability between samples of Canadian and Irish participants

### SF-HDQ scoring algorithms

**Fig. 3:**
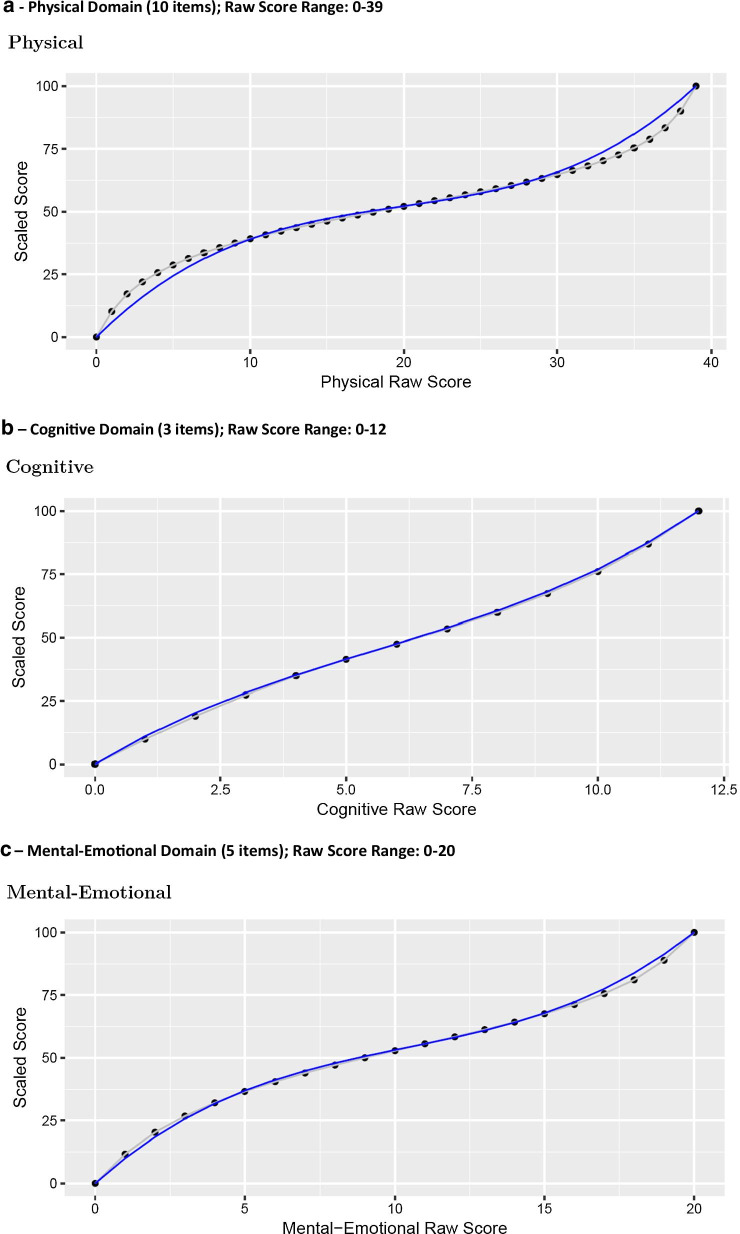

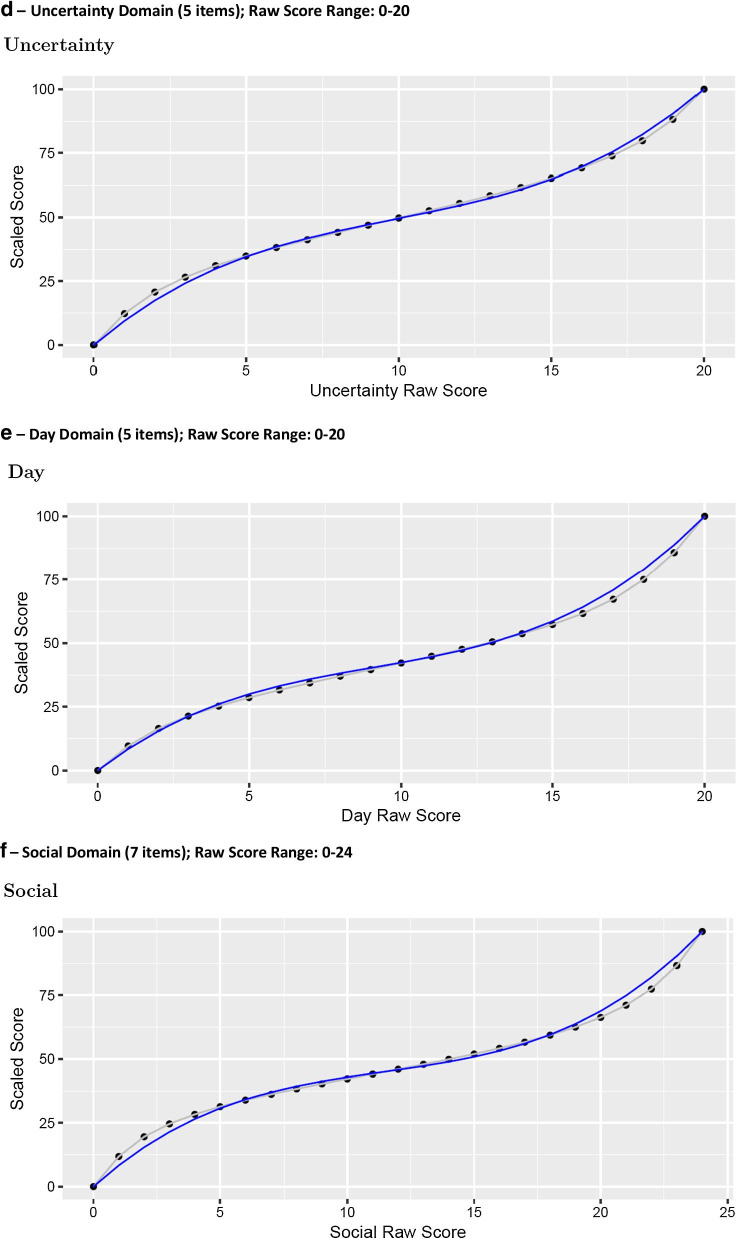
Cubic models fitting raw SF-HDQ summary scores with scaled Rasch scores

Graphs in Fig. 3 (Fig. 3a–f) illustrate the person total sum score (total) against the Rasch person score, rescaled from logits to a score from 0 to 100 (grey line) for each domain. The blue line in Fig. 3 is the fitted (predicted) curve from the cubic function fit to those scores, scaled from 0 to 100.

Tables 8, 9, 10, 11, 12 and 13 includes the Rasch-based, model predicted person score estimates for each value of a raw domain summed score for all six domains. We included predicted estimates for the original scale and the model predicted Rasch person-score on the 0–100 interval scale.

**Table 8:** Conversation table - Physical domain (10 items)—raw summed scores to predicted Rasch person scores for SF-HDQ physical domain

Physical domain (10 items)		
Raw summed score—total Range: 0–39	Model predicted person score (location)	Model predicted person score (0–100 scale)
0	− 4.075	0
1	− 3.261	6
2	− 2.703	11
3	− 2.321	16
4	− 2.026	20
5	− 1.783	24
6	− 1.575	28
7	− 1.393	31
8	− 1.230	34
9	− 1.083	37
10	− 0.948	39
11	− 0.823	41
12	− 0.705	43
13	− 0.594	45
14	− 0.489	46
15	− 0.387	47
16	− 0.289	48
17	− 0.194	49
18	− 0.101	50
19	− 0.009	51
20	0.082	52
21	0.173	53
22	0.264	54
23	0.356	55
24	0.450	56
25	0.546	57
26	0.645	59
27	0.748	60
28	0.856	62
29	0.970	64
30	1.093	66
31	1.226	68
32	1.371	71
33	1.535	74
34	1.722	77
35	1.943	81
36	2.215	85
37	2.573	90
38	3.107	95
39	3.903	100

**Table 9:** Conversation table - Cognitive domain (3 items)—raw summed scores to predicted Rasch person scores for SF-HDQ cognitive domain

Cognitive domain (3 items)		
Raw summed score—total Range: 0–12	Model predicted person score (location)	Model predicted person score (0–100 scale)
0	− 5.086	0
1	− 4.038	11
2	− 3.069	20
3	− 2.175	28
4	− 1.367	35
5	− 0.675	42
6	− 0.036	48
7	0.601	54
8	1.286	61
9	2.083	68
10	3.013	77
11	4.156	87
12	5.560	100

**Table 10:** Conversation table - Mental-Emotional domain (5 items)—raw summed scores to predicted Rasch person scores for SF-HDQ mental-emotional domain

Mental-emotional domain (5 items)		
Raw summed score—total Range: 0–20	Model predicted person score (location)	Model predicted person score (0–100 scale)
0	− 3.997	0
1	− 3.098	10
2	− 2.428	18
3	− 1.930	26
4	− 1.522	32
5	− 1.174	37
6	− 0.872	41
7	− 0.604	45
8	− 0.361	48
9	− 0.135	51
10	0.082	53
11	0.295	56
12	0.509	58
13	0.730	61
14	0.964	64
15	1.219	68
16	1.505	72
17	1.841	78
18	2.264	84
19	2.867	91
20	3.726	100

**Table 11:** Conversation table Uncertainty domain (5 items)—raw summed scores to predicted Rasch person scores for SF-HDQ uncertanity domain

Uncertainty domain (5 items)		
Raw summed score—total Range: 0–20	Model predicted person score (location)	Model predicted person score (0–100 scale)
0	− 3.683	0
1	− 2.779	9
2	− 2.155	17
3	− 1.725	24
4	− 1.393	30
5	− 1.114	35
6	− 0.868	39
7	− 0.643	42
8	− 0.431	45
9	− 0.224	47
10	− 0.020	50
11	0.187	52
12	0.398	55
13	0.620	57
14	0.857	61
15	1.117	65
16	1.411	70
17	1.758	75
18	2.194	82
19	2.811	90
20	3.683	100

**Table 12:** Conversation table - Day-to-Day Activities domain (5 items)—raw summed scores to predicted Rasch person scores for SF-HDQ day-to-day activities domain

Day domain (5 items)		
Raw summed score—total Range: 0–20	Model predicted person score (location)	Model predicted person score (0–100 scale)
0	− 3.973	0
1	− 3.103	8
2	− 2.484	15
3	− 2.043	21
4	− 1.690	26
5	− 1.388	30
6	− 1.117	33
7	− 0.867	36
8	− 0.627	38
9	− 0.394	40
10	− 0.160	42
11	0.079	45
12	0.327	47
13	0.591	50
14	0.879	54
15	1.205	59
16	1.596	64
17	2.102	71
18	2.810	79
19	3.769	89
20	5.066	100

**Table 13:** Conversation table Social domain (7 items)—raw summed scores to predicted Rasch person scores for SF-HDQ social domain

Social domain (7 items)		
Raw summed score—total Range: 0–24	Model predicted person score (location)	Model predicted person score (0–100 scale)
0	− 3.509	0
1	− 2.627	8
2	− 2.051	15
3	− 1.675	21
4	− 1.397	27
5	− 1.172	31
6	− 0.980	34
7	− 0.810	37
8	− 0.653	39
9	− 0.505	41
10	− 0.362	43
11	− 0.222	44
12	− 0.081	46
13	0.062	47
14	0.209	49
15	0.363	51
16	0.529	53
17	0.710	56
18	0.914	59
19	1.150	64
20	1.434	69
21	1.791	75
22	2.264	82
23	2.951	90
24	3.948	100

Additional file 4 provides the scoring algorithms for each domain that will yield simple Rasch-based interval SF-HDQ domain scores (range: 0–100) with higher scores indicating greater severity of disability.

## Discussion

The Short-Form HIV Disability Questionnaire (SF-HDQ) is comprised of 35 items (reduced from the original 69-items) spanning six domains: physical (10 items), cognitive (3 items), mental-emotional (5 items), uncertainty (5 items), day-to-day activities (5 items), and social (7 items). Each domain yields an interval scale score derived from the Rasch model ranging from 0 to 100, with higher scores indicating greater severity of disability. Among the final 35 items, 3 were reordered: 1 item in the physical domain was rescored to 4 categories (HDQ3); and 2 items in social domain (HDQ63 and HDQ67) were rescored to 3 categories to result in ordered categories. All remaining items retained original five categories ranging from no challenge (0) to extreme difficulty (4).

Decisions to retain or remove items in the final SF-HDQ model required consideration of the Rasch model results in combination with clinical relevance of items. The development of the SF-HDQ involved multiple iterations to determine ideal fit that considered a combination of model fit indices, item fit indices (fit residuals), extent to which an item may be captured conceptually by another item in the domain, and clinical importance (Additional file 1). All but one domain met pre-specified criteria for model fit, demonstrated by domain Cronbach’s alphas and PSIs ≥ 0.70 with the exception of difficulties with day-to-day activities domain (PSI = 0.69) (Tables 2, 3, 4, 5, 6, 7). We considered this acceptable given the proximity to the threshold of model fit, and this model demonstrated ideal unidimensionality over other model iterations with PSIs ≥ 0.70 (Additional file 1). While three items in the final model (HDQ53, HDQ35, HDQ22) demonstrated significance for fit residuals, the absolute residual value was < 2.5, hence we retained them in the model. Two items (HDQ21, HDQ68) possessed significance for fit residuals > 2.5 or < − 2.5. We retained HDQ21 due to clinical importance [53, 54] and the need for a minimum of three items in the cognitive domain in order to comprise a latent variable [47, 48]. HDQ68 (tend to isolate self) may be referring to a ‘strategy’ that may lead to challenges to social inclusion rather than comprise the concept of social inclusion itself. Nevertheless, we retained due to clinical importance [55–58], and if removed from the model, it worsened fit (Tables 2, 3, 4, 5, 6, 7).

DIF for country resulted in three items in the social domain (HDQ63: housing; HDQ65: find it hard to ask others for help; HDQ69: find it hard to take part in leisure or recreational activities), which we retained due to their clinical importance (Table 7). DIF for country may be explained by the cultural differences that may exist between the Canadian and Irish participants and the willingness for participants to disclose their susceptibility to chronic illness and challenges living with HIV [59]. Differences in societal structures and diversity across health system settings can influence experiences and interpretations of disability. Future cross-cultural assessment of the SF-HDQ across countries will be important for determining international utility of the SF-HDQ in clinical and community-based practice [60, 61].

Each domain represents a dimension of disability, with its own domain scores that collectively describe the larger construct of disability. The scoring algorithms (Additional file 4) provide the opportunity to automatically generate domain scores with electronic administration using scoring software so that researchers, clinicians and adults living with HIV can obtain domain scores immediately upon completion. The scoring conversion charts (Tables 8, 9, 10, 11, 12, 13) will allow clinicians who administer the SF-HDQ using paper-based methods in clinic to easily convert the raw summed domain score to the Rasch interval scale score (0–100). This, with the reduced length of the questionnaire, will enhance the clinical utility of the SF-HDQ.

### Implications for practice and research

This study is the first to establish a PROM to assess the multi-dimensional nature of disability among adults aging with HIV. By retaining the six domain structure of the SF-HDQ, the PROM builds on the previously validated *Episodic Disability Framework* and HDQ with adults living with HIV [17, 19, 24, 25, 32]. Results will help to advance instrumentation and methods for PROM implementation to enhance feasibility, relevance and ease of use in clinical practice. For individuals aging with multimorbidity and complex health needs, PROMs should be embedded in individuals’ personalized needs and goals for care [62]. Standardized PROMs that capture the nature, fluctuation and extent of disability are critical to identify health priorities, to guide timely and appropriate care, and to evaluate the effectiveness of interventions for those aging with HIV [63–65]. The SF-HDQ has the potential to enhance person-provider communication, and identify an individual’s needs enhancing overall person-centered care [66, 67]. HIV-specific PROMs such as the SF-HDQ are particularly important for HIV care as it goes beyond traditional outcome measures of viral load or survival to describe person-centred outcomes (mental health, social inclusion, uncertainty) and their change over time, enhance communication by empowering adults aging with HIV to articulate their health challenges and needs, facilitate goal-setting, and guide referrals to available services. At the service delivery level, the SF-HDQ may help clinics or community-based organizations to better understand the changing needs of individuals as they age with HIV, evaluate the impact of interventions, programs and models of service delivery, and inform areas of resource allocation for future programming and service provision [68].

Our Rasch analysis focused on the severity scale of the HDQ building on our earlier work using exploratory and confirmatory factor analysis, and structural equation modeling to establish the domain structure and validate the six latent constructs that comprise disability in the HDQ, and examine relationships between dimensions of disability [25, 32, 52]. This work directly addresses one of the seven key research priorities in HIV, aging and rehabilitation to advance the development and use of PROMs in HIV and aging [69]. While our aim developing the SF-HDQ is to facilitate uptake for use in clinical and community-based practice, the SF-HDQ also may reduce respondent burden in research studies involving multiple PROMs administered in combination. Next steps include refinement of the SF-HDQ, such as minor wording revisions, questionnaire instructions, response option categories for the three reordered items, and the numbering (and order) of SF-HDQ items.

Our approach is not without limitations. Only 18% of the sample were women, which is below the estimated prevalence of women living with HIV in Canada (23%) and Ireland (29%) [70, 71], and few participants identified as trans or two spirited limiting our DIF analysis for gender. Furthermore, the difference in sample sizes across the Canadian and Irish samples (almost 10:1 ratio) may have increased the probability of type I error as well as reduced the power of DIF detection [72, 73]. The SF-HDQ was derived from adults aging with HIV in high income countries. While validation work is underway in South Africa with the original HDQ, future examination of the properties of the SF-HDQ with adults living with HIV in low or middle-income contexts is warranted. Future work should include measurement property assessment including cross-cultural validity of the SF-HDQ for use with adults living with HIV in countries where there may be different cultural perspectives of disability related to country. Lastly, our Rasch analysis was limited to cross-sectional HDQ data. Work is underway to examine the consistency of SF-HDQ scores with the Rasch model over time using long-form HDQ data with a sample of adults living with HIV engaged in an exercise intervention study in Canada [74].

## Conclusions

The newly proposed 35-item SF-HDQ offers a brief yet comprehensive patient-reported outcome measure of disability to describe the nature and extent of disability experienced by adults living with HIV. The scoring algorithm offers a feasible way to convert raw summed domain scores to the Rasch-based score on an interval level scale ranging from 0 to 100. This shortened questionnaire and its new scoring algorithm may be used by clinicians, researchers, and other care providers working in busy clinical and community-based settings as a tool to comprehensively measure disability and to better identify the health-related needs of adults living with HIV.

## Supplementary Information

**Additional file 1** Summary of Fit Statistics for the Initial to Final Models of the SF-HDQ Domains.

**Additional file 2** Item Category Probability Curves for SF-HDQ Items (n=35 items).

**Additional file 3** Overview of Original HDQ Items and Final SF-HDQ Items.

**Additional file 4** Scoring Algorithms for SF-HDQ Domains.

## Data Availability

The datasets used and analyzed during this study may be available by request to the corresponding author on reasonable request.

## Abbreviations

DIF: Differential item functioning
HIV: Human Immunodeficiency Virus
HDQ: HIV Disability Questionnaire
PCA: Principal components analysis
PROM: Patient-reported outcome measure
PSI: Person Separation Index/Indices
SF-HDQ: Short-form HIV Disability Questionnaire
