# Supplementary material for: Developing a short-form version of the HIV Disability Questionnaire (SF-HDQ) for use in clinical practice: a Rasch analysis
Source: Health Qual Life Outcomes. 2021 Jan 6;19:6. doi: 10.1186/s12955-020-01643-2 (PMC7789190; doi:10.1186/s12955-020-01643-2)
Supplement: Supplementary file 1 — Additional file 1 Summary of Fit Statistics for the Initial to Final Models of the SF-HDQ Domains. [file 12955_2020_1643_MOESM1_ESM.pdf]

Additional File 1 – Summary of Fit Statistics for the Initial to Final Model of the SF-HDQ Domains

**Model**	**Description**	**Number of Items**	**Sample Size**	**Item Thresholds Ordered**	**X^2 Fit Statistic (degrees of freedom; p value)**<br>***Ideal not significant***	**Cronbach's alpha**<br>***Ideal ≥0.70***	**Person Separation Index (PSI)**<br>***Ideal ≥0.70***	**Items with Fit Residuals (>+/-2.5) with significance after Bonferroni adjustment (0.05/# items in original domain) Item (Fit residual)**	**Unidimensionality (Proportion of t-tests significant)**<br>***Ideal ≤5%***	**Differential Item Functioning (DIF)**
**PHYSICAL DOMAIN**										
Initial Model 1	All 20 items in the original domain	20 items	1010	3 items disordered HDQ3, HDQ8, HDQ15	476 (df:180; p=0.00)	0.903	0.869	Not assessed	Not assessed	Not assessed
Model 5	Rescored HDQ3[a]<br>HDQ8[a]<br>Deleted HDQ15 because rescoring nonsensical	19 items	1010	All items ordered	511 (df:171; P<0.001)	0.899	0.867	4 items<br>HDQ9 (9.71)<br>HDQ12 (-3.32)<br>HDQ13 (-3.174)<br>HDQ8 (-2.64)<br>HDQ2 (3.68)[c]	Not assessed	Not assessed
Model 10	Deleted<br>HDQ9<br>HDQ2<br>HDQ12<br>HDQ8<br>HDQ13	14 items	999	All items ordered	196 (df:126; P<0.001)	0.877	0.827	None	3.1%	No DIF
Added HDQ13 (muscle cramps) back into model because this item was not correlated highly with aches or pains (may be distinct) but not similar enough to merge with HDQ14 I have stomach cramps.										
Model 13	Deleted<br>HDQ18<br>HDQ19<br>HDQ16	12 items	982	All items ordered	179 (df: 108; p<0.001)	0.872	0.818	HDQ17 (3.08)[c] (trouble sleeping) retained because item considered clinically important.	0.61%	No DIF
Model 18 FINAL	Deleted<br>HDQ14<br>HDQ6<br>HDQ16	10 items	981	All items ordered	154 (df: 90; p<0.001)	0.848	0.790	None	0.41%	No DIF

Additional File 1 – Summary of Fit Statistics for the Initial to Final Model of the SF-HDQ Domains

Model	Description	Number of Items	Sample Size	Item Thresholds Ordered	X^2 Fit Statistic (degrees of freedom; p value)<br>*Ideal not significant*	Cronbach's alpha<br>*Ideal ≥0.70*	Person Separation Index (PSI)<br>*Ideal ≥0.70*	Items with Fit Residuals (>+/-2.5) with significance after Bonferroni adjustment (0.05/# items in original domain)<br>Item (Fit residual)	Unidimensionality (Proportion of t-tests significant)<br>*Ideal ≤5%*	Differential Item Functioning (DIF)
**COGNTIIVE DOMAIN**										
Initial & Final	No changes to 3 items in original domain	3 items	730	All items ordered	76 (df: 21; p<0.001)	0.710	0.776	HDQ21 (2.76)[i]	1.23%	No DIF
**MENTAL-EMOTIONAL DOMAIN**										
Initial Model 1	All 11 items in original domain	11 items	967	All items ordered	360 (df: 99; p<0.001)	0.928	0.891	HDQ28 (10.90)<br>HDQ33 (-5.07)<br>HDQ29 (-5.00)<br>HDQ30 (-3.81)<br>HDQ34 (3.07)	3.93%	HDQ34 (country)
Model 6	Deleted<br>HDQ28<br>HDQ33<br>HDQ29<br>HDQ34<br>HDQ30	6 items	918	All items ordered	67 (df: 54: p=0.11)[c]	0.868	0.810	HDQ31 (3.02)[c]	1.63%	No DIF
Model 7 FINAL	Deleted HDQ31 (large residual although not statistically significant; but determined not clinically important)	5 items	903	All items ordered	54 (df: 45; p=0.17)[d]	0.849	0.792	None	1.14%	No DIF

Additional File 1 – Summary of Fit Statistics for the Initial to Final Model of the SF-HDQ Domains

Model	Description	Number of Items	Sample Size	Item Thresholds Ordered	X^2 Fit Statistic (degrees of freedom; p value) *Ideal not significant*	Cronbach's alpha *Ideal ≥0.70*	Person Separation Index (PSI) *Ideal ≥0.70*	Items with Fit Residuals (>+/-2.5) with significance after Bonferroni adjustment (0.05/# items in original domain) Item (Fit residual)	Unidimensionality (Proportion of t-tests significant) *Ideal ≤5%*	Differential Item Functioning (DIF)
**UNCERTAINTY DOMAIN**										
Initial Model 1	All 14 original items in the model	14 items	995	5 items disordered HDQ42 HDQ43 HDQ45 HDQ47 HDQ48	544 (df: 126; p<0.001)	0.918	0.899	Not assessed	Not assessed	Not assessed
Model 12	Rescored[a] HDQ43 HDQ45 Deleted HDQ42 HDQ47 HDQ48 as rescoring nonsensical	11 items	977	All items ordered	381 (df: 99; p<0.001)	0.906	0.885	HDQ46 (7.64) HDQ44 (6.91) HDQ37 (-5.53) HDQ38 (-4.71) HDQ35 (-4.61) HDQ36 (-2.66)	9.72%	Not assessed
Model 15	Deleted HDQ46 HDQ44 HDQ45 (largest residual)	8 items	947	All items ordered	235 (df: 72; p<0.001)	0.896	0.871	HDQ40 (5.21) HDQ43 (4.56) HDQ37 (-5.01) HDQ38 (-3.56) HDQ35 (-2.60)	2.53%	HDQ40 (country) (likely attributed to more Irish working compared with CAD participants)
Model 17	Deleted HDQ40 HDQ37	6 items	931	All items ordered	84 (df: 54; p=0.006)	0.860	0.817	HDQ38 (-3.19)	2.26%	No DIF
Model 18	Deleted HDQ38	5 items	925	All items ordered	75 (df: 45; p=0.003)	0.818	0.761	None	1.62%	No DIF
Model 19	Added HDQ38 back into model Deleted HDQ43	5 items	921	All items ordered	58 (df: 45; p=0.09)[d]	0.844	0.796	None	2.17%	No DIF

Additional File 1 – Summary of Fit Statistics for the Initial to Final Model of the SF-HDQ Domains

Model	Description	Number of Items	Sample Size	Item Thresholds Ordered	X^2 Fit Statistic (degrees of freedom; p value) *Ideal not significant*	Cronbach's alpha *Ideal ≥0.70*	Person Separation Index (PSI) *Ideal ≥0.70*	Items with Fit Residuals (>+/-2.5) with significance after Bonferroni adjustment (0.05/# items in original domain) Item (Fit residual)	Unidimensionality (Proportion of t-tests significant) *Ideal ≤5%*	Differential Item Functioning (DIF)
We went back to Model 15 (8 items) to explore an alternate 5 item combination for better model fit.										
Model 23	Deleted HDQ37 HDQ43 not considered as clinically relevant in current state of combination ART	6 items	936	All items ordered	97 (df: 54; p<0.001)	0.860	0.825	None	0.85%	HDQ40 (country) Significant DIF but not >1.0 logit difference (likely attributed to more Irish working compared with CAD participants.
Model 24 FINAL	Deleted HDQ38	5 items	930	All items ordered	59 (df: 45: p=0.10)[d]	0.823	0.780	None	1.72%	HDQ40 (country) >1.0 logit difference and item considered clinically important (likely attributed to more Irish working compared with CAD participants)
**DIFFICULTIES WITH DAY-TO-DAY ACTIVITIES DOMAIN**										
Initial Model 1	All 9 original items in the model	9 items	753	All items ordered	295 (df: 81; p<0.001)	0.881	0.796	HDQ56 (8.02)[e] HDQ52 (-4.79) HDQ55 (-4.50) HDQ53 (-3.44)[c]	3.85%	Not assessed
Model 3	Deleted HDQ56[e] HDQ52	7 items	701	All items ordered	81 (df: 63; p=0.06)[c]	0.857	0.775	None	5.28%	No DIF
Model 4	Deleted HDQ51	6 items	679	All items ordered	105 (df: 54; p<0.001)	0.833	0.735	None	5.15%	No DIF
Model 9 - FINAL	Deleted HDQ57[f]	5 items	675	All items ordered	77 (df: 45; p=0.002)	0.795	0.690	None	0.74%	No DIF

Additional File 1 – Summary of Fit Statistics for the Initial to Final Model of the SF-HDQ Domains

Model	Description	Number of Items	Sample Size	Item Thresholds Ordered	X^2 Fit Statistic (degrees of freedom; p value) *Ideal not significant*	Cronbach's alpha *Ideal ≥0.70*	Person Separation Index (PSI) *Ideal ≥0.70*	Items with Fit Residuals (>+/-2.5) with significance after Bonferroni adjustment (0.05/# items in original domain) Item (Fit residual)	Unidimensionality (Proportion of t-tests significant) *Ideal ≤5%*	Differential Item Functioning (DIF)
**SOCIAL DOMAIN**										
Initial Model 1	All 12 items in the model	12 items	966	2 items disordered HDQ63 HDQ67	337 (df: 108; p<0.001)	0.903	0.862	HDQ67 (6.22)<br>HDQ68 (-5.18)<br>HDQ66 (-5.07)<br>HDQ64 (4.96)<br>HDQ59 (-3.77)	Not assessed	Not assessed
Model 4	Rescored HDQ63[b] HDQ67[b]	12 items	966	All items ordered	306 (df: 108; p<0.001)	0.898	0.862	HDQ64 (6.06)<br>HDQ68 (-4.17)<br>HDQ66 (-4.32)<br>HDQ59 (-3.92)	Not assessed	Not assessed
Model 7	Deleted HDQ64 HDQ59 HDQ66	9 items	986	All items ordered	182.24 (df: 81; p<0.001)	0.856	0.806	HDQ60 (-2.70)<br>HDQ68 (-2.59)	4.18%	HDQ65 (country)<br>HDQ67 (country)
Model 16 - FINAL	Deleted HDQ60 HDQ61	7 items	943	All items ordered	87 (df: 63; p=0.03)	0.794	0.745	HDQ68 (-3.14)[g]	1.27%	HDQ63 (country)[h]<br>HDQ65 (country)[h]<br>HDQ69 (country)[h]

Notes: Sample size varied as we omitted extreme values from model analyses.

[a] Items rescored to 4 categories;

[b] Items rescored to 3 categories;

[c] Item has absolute F value fit residual >2.5, but not significant with Bonferroni adjustment for that domain. Items listed in order of descending absolute F value

[d] Model fit X^2 test - p value not significant (indication of model fit) – achieved for mental-emotional and uncertainty domains;

[e] HDQ56 (trouble keeping track of finances) – deleted this item; item severity may be due to item interpreted as financial insecurity rather than ability to keep track of finances as daily activity as intended by the item;

[f] HDQ57 (trouble getting around, such as driving or taking public transportation) - Deleted because large DIF in graph analysis for country and this item could be considered captured with HDQ55 (trouble getting out to do errands) because one may need to get around in order to conduct errands;

[g] HDQ68 (tend to isolate self from others) retained because subsequent model with this item deleted demonstrated worse fit (lower PSI);

[h] DIF for country (HDQ63: housing; HDQ65: find it hard to ask others for help; HDQ69: find it hard to take part in leisure or recreational activities). Items retained given clinical importance and expected cultural differences between samples of Canadian and Irish participants ;

[i] HDQ21 – Significant Residual >2.5: retained this item because the cognitive domain has a minimum of 3 items.
