# Supplementary material for: Developing a short-form version of the HIV Disability Questionnaire (SF-HDQ) for use in clinical practice: a Rasch analysis
Source: Health Qual Life Outcomes. 2021 Jan 6;19:6. doi: 10.1186/s12955-020-01643-2 (PMC7789190; doi:10.1186/s12955-020-01643-2)

Additional File 2– Item Category Probability Curves for SF-HDQ Items (n=35 items)

Physical Domain (10 items)

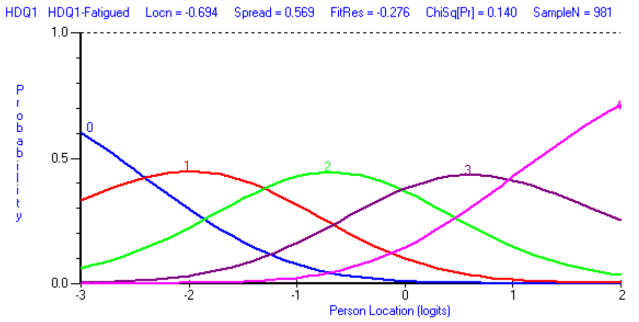

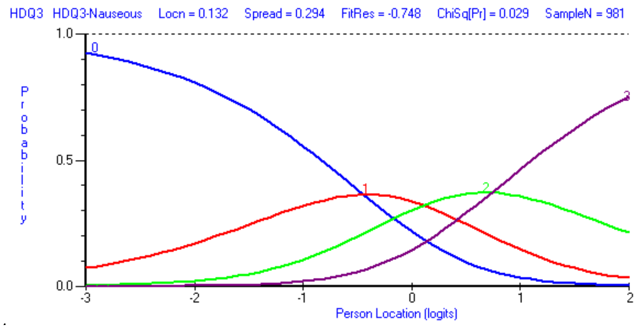

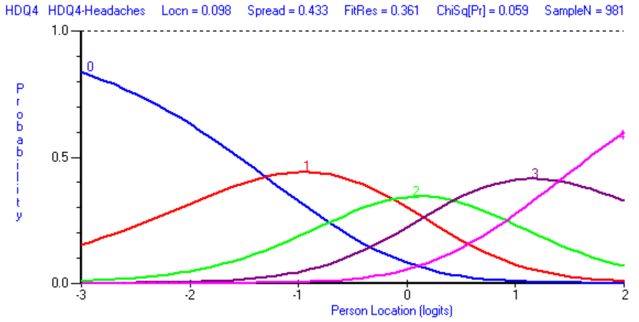

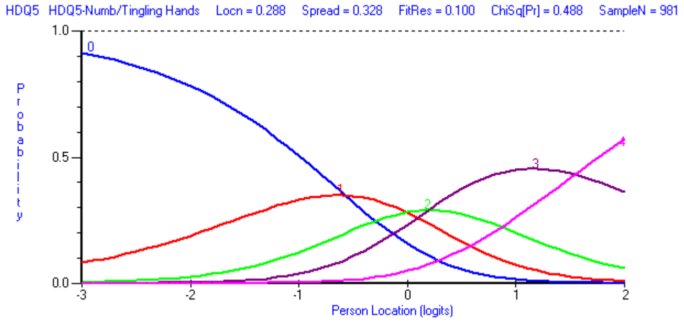

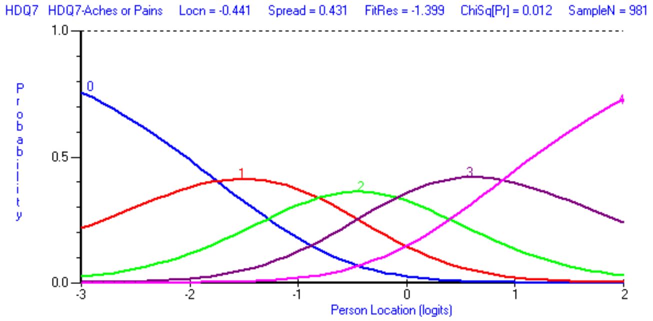

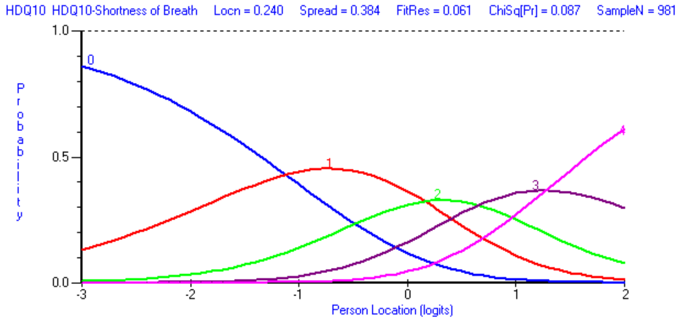

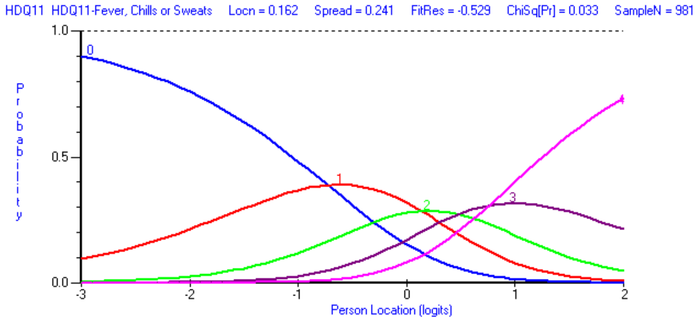

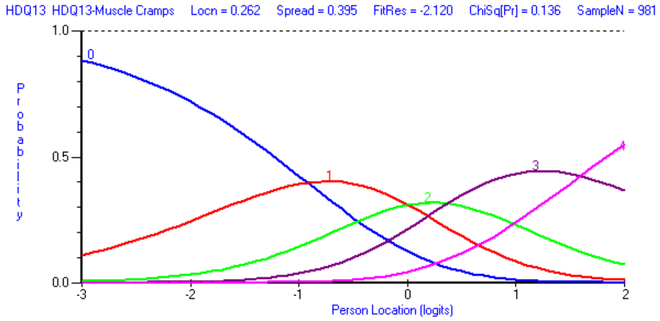

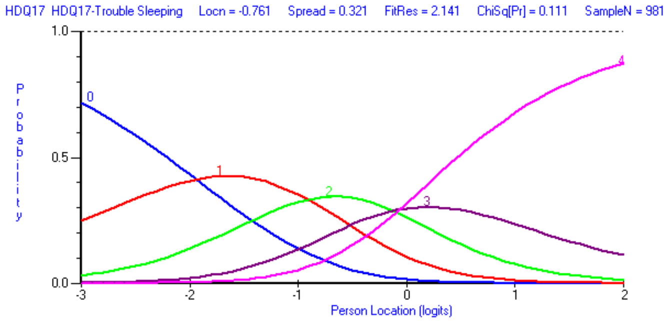

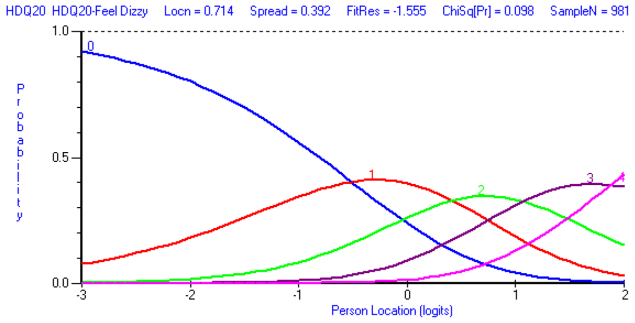

Additional File 2– Item Category Probability Curves for SF-HDQ Items (n=35 items)

Cognitive Domain (3 items)

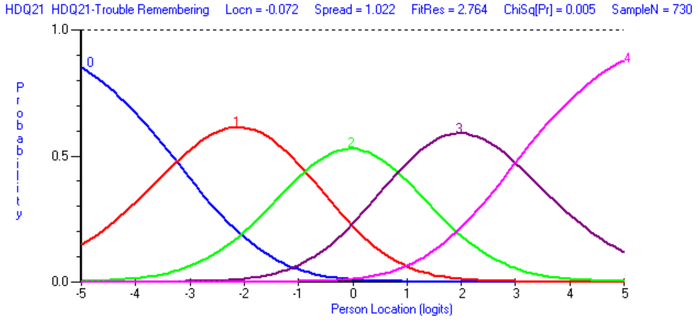

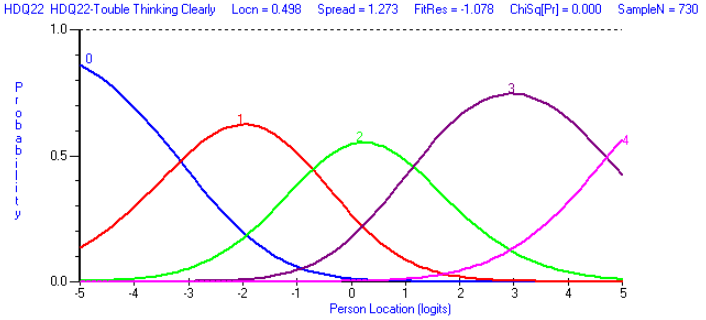

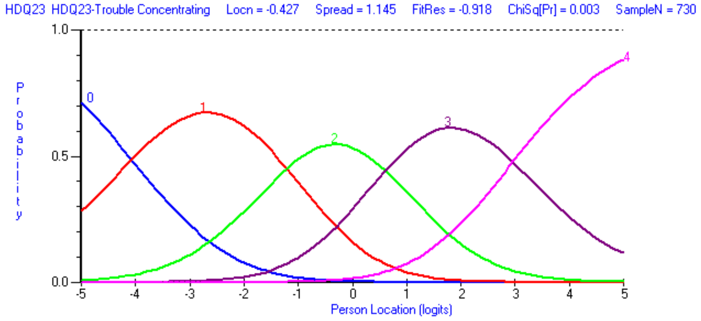

Mental-Emotional Domain (5 items)

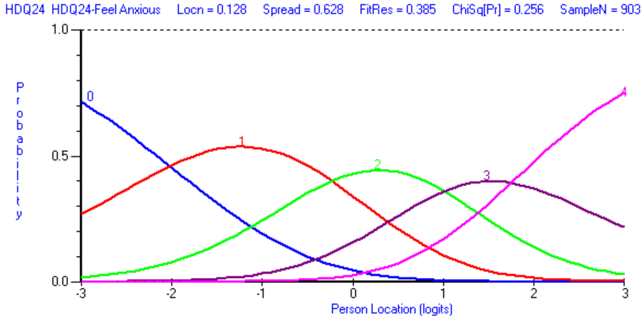

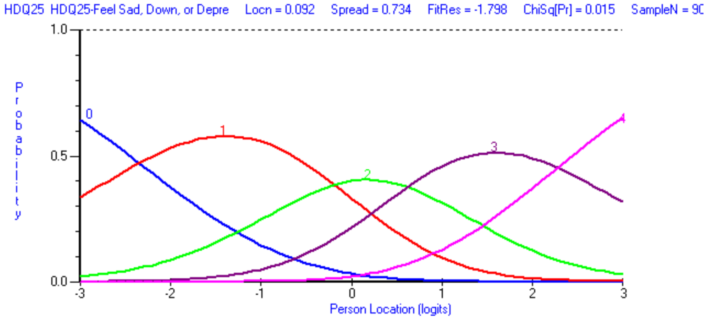

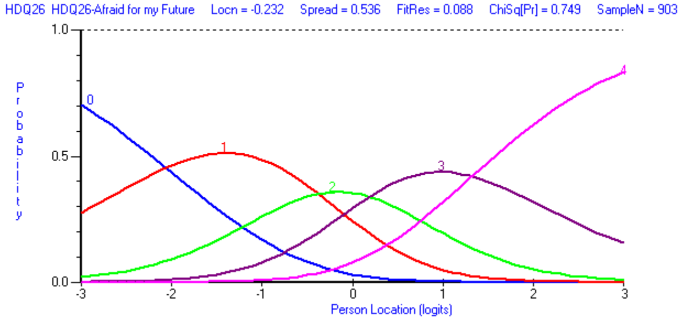

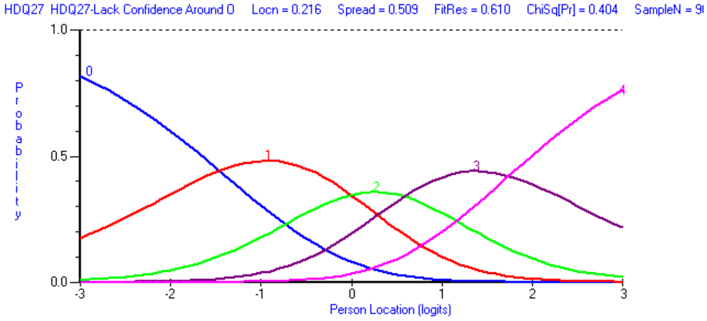

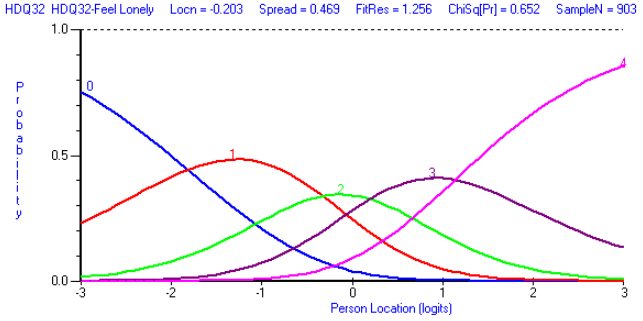

Additional File 2– Item Category Probability Curves for SF-HDQ Items (n=35 items)

Uncertainty Domain (5 items)

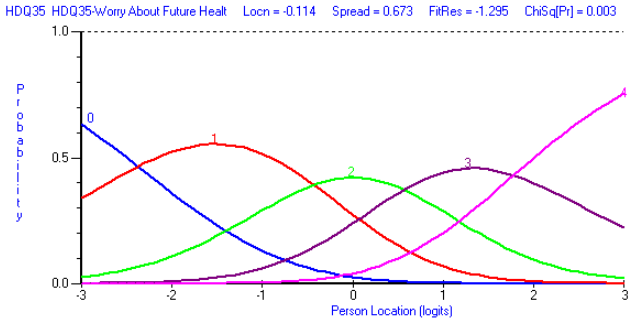

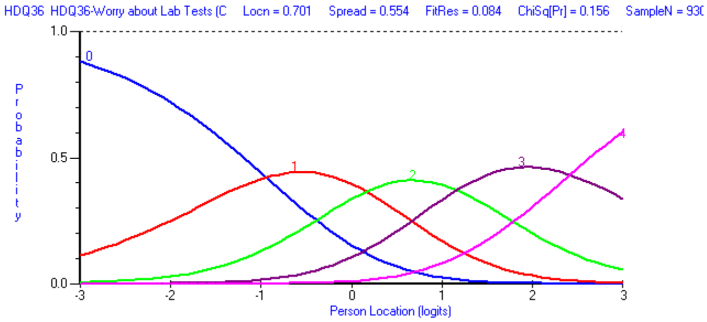

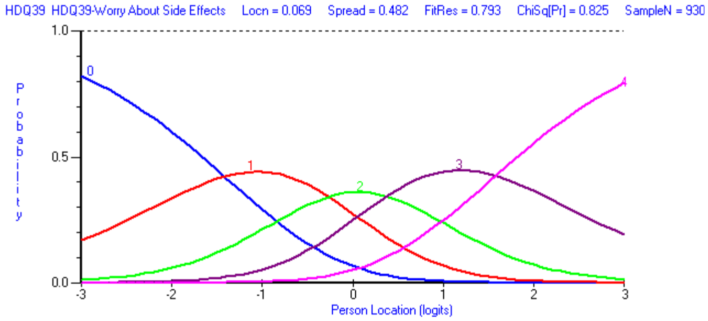

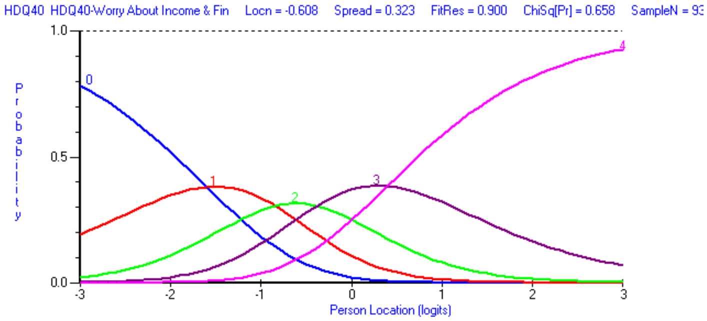

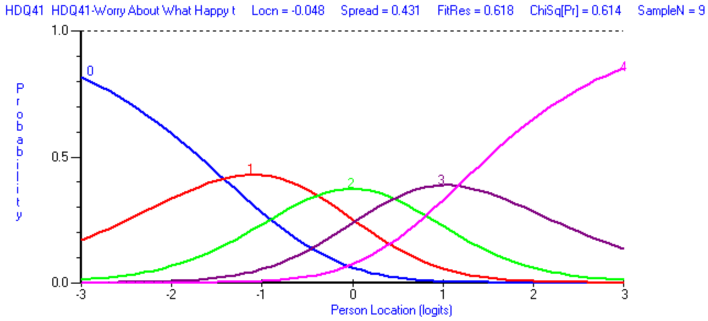

Day-to-Day Activities Domain (5 items)

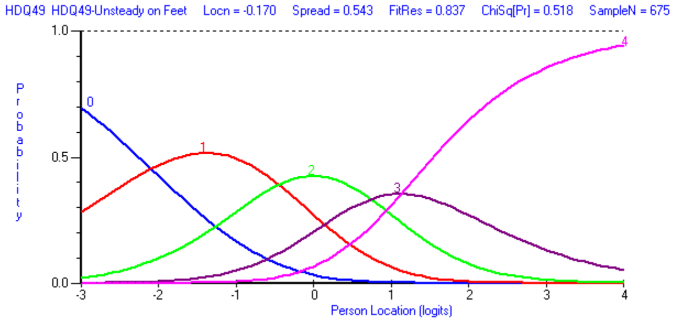

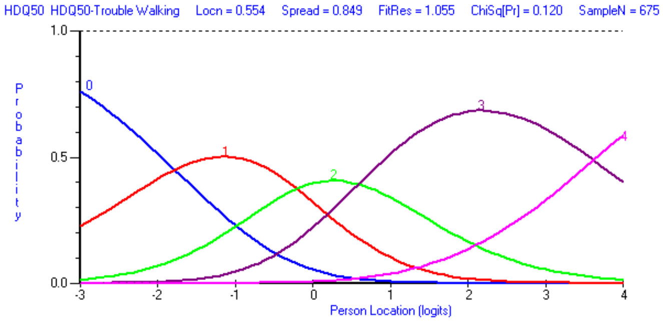

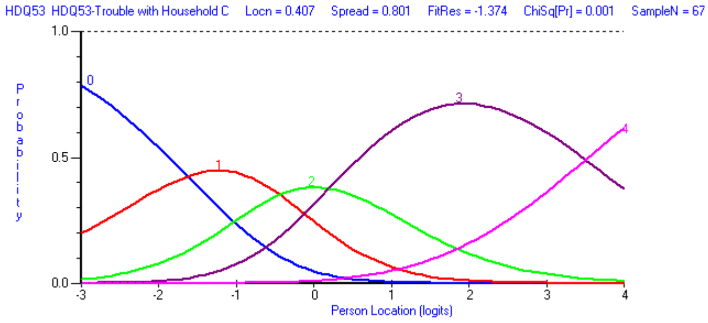

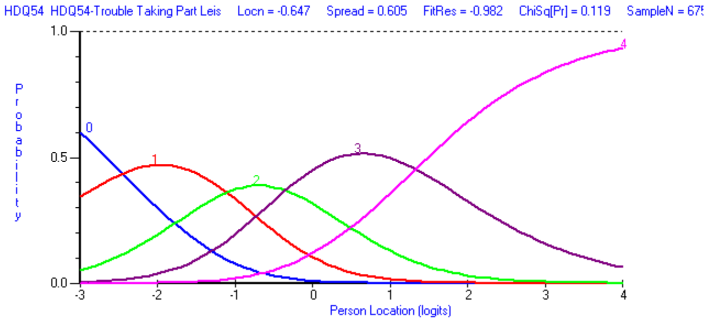

Additional File 2– Item Category Probability Curves for SF-HDQ Items (n=35 items)

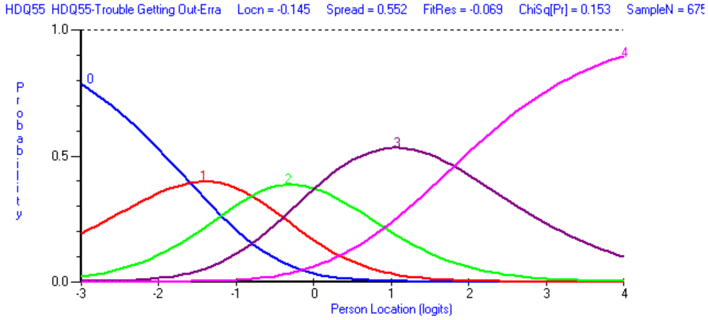

Social Domain (7 items)

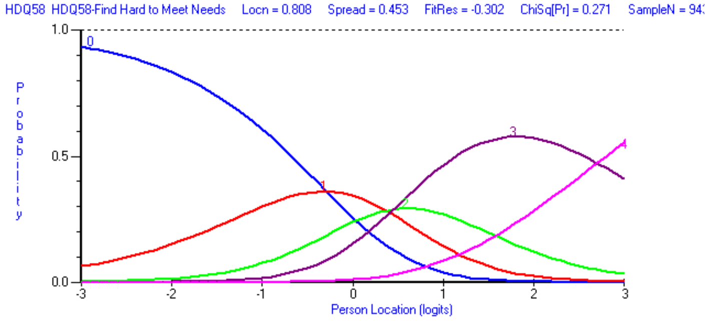

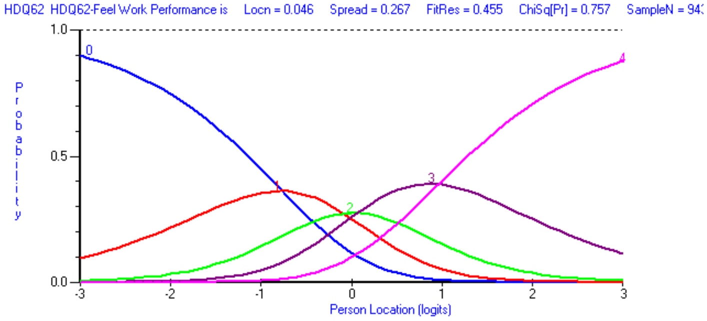

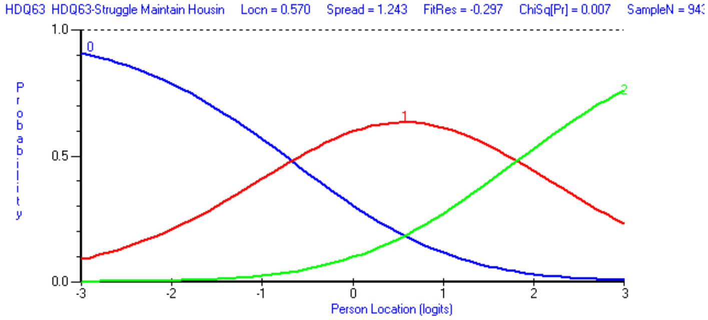

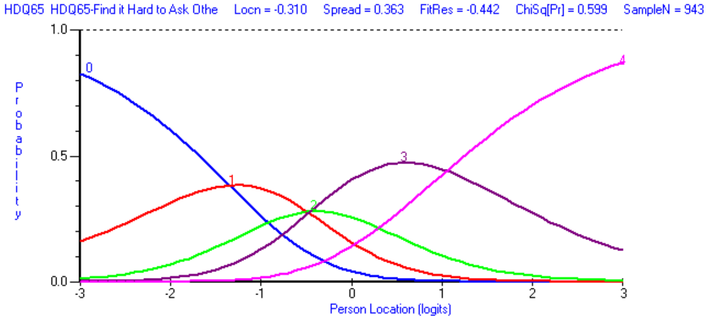

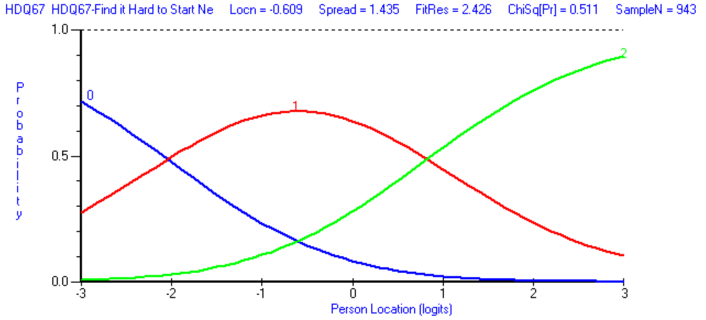

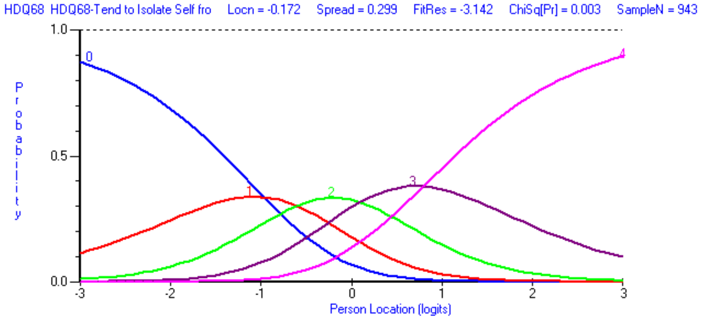

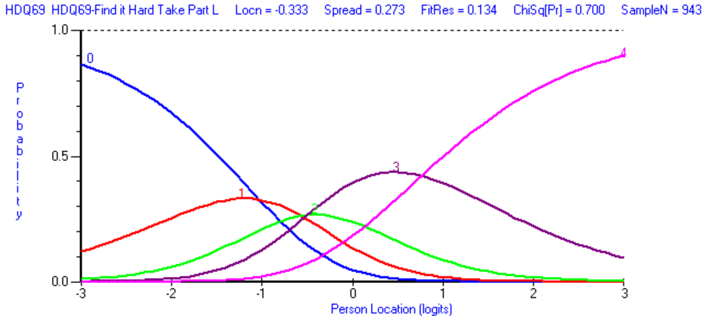

Supplement: Supplementary file 2 — Additional file 2 Item Category Probability Curves for SF-HDQ Items (n=35 items). [file 12955_2020_1643_MOESM2_ESM.pdf]
