# Supplementary material for: Developing a short-form version of the HIV Disability Questionnaire (SF-HDQ) for use in clinical practice: a Rasch analysis
Source: Health Qual Life Outcomes. 2021 Jan 6;19:6. doi: 10.1186/s12955-020-01643-2 (PMC7789190; doi:10.1186/s12955-020-01643-2)
Supplement: Supplementary file 3 — Additional file 3 Overview of Original HDQ Items and Final SF-HDQ Items. [file 12955_2020_1643_MOESM3_ESM.pdf]

Additional File 3 – Original HDQ Items (n=69) and Final SF-HDQ Items (n=35)

**Physical Domain**		
**Item #**	**Original HDQ Items**	**Final SF-HDQ Items**
1	Fatigue	Fatigue
2	Diarrhea	--
3	Nausea	Nausea *(Rescored to 4 response categories (0-3))*
4	Headaches	Headaches
5	Numbness or tingling in hands	Numbness or tingling in hands *or feet*
6	Numbness or tingling in feet	--
7	Aches or pains.	Aches or pains
8	Trouble swallowing food	--
9	Decreased libido	--
10	Shortness of breath	Shortness of breath
11	Fever, chills, or sweats	Fever, chills, or sweats
12	Muscle weakness	--
13	Muscle cramps	Muscle cramps
14	Stomach cramps	--
15	Losing weight	--
16	Lack an appetite for food.	--
17	Trouble sleeping	Trouble sleeping
18	Vision problems	--
19	Hearing problems	--
20	Feel dizzy	Feel dizzy
**Physical Total Number of Items**	**20 items**	**10 items**
**Notes:** In future refinement of the SF-HDQ, we will merge HDQ5 and HDQ6 to one item: "I have numbness or tingling in hands or feet" given similar neuropathic source; retained HDQ5 over HDQ6 in the model due to better fit (see Additional File 1); HDQ13 (muscle cramps) and HDQ14 (stomach cramps) clinically distinct, hence items not merged. Retained muscle cramps because item not highly correlated with aches or pains (suggesting it is distinct) (see Additional file 1).		

**Cognitive Domain**		
**Item #**	**Original HDQ Items**	**Final SF-HDQ Items**
21	Trouble remembering like appointments and when to take medication	Trouble remembering like appointments and when to take medication
22	Trouble thinking clearly	Trouble thinking clearly
23	Trouble concentrating	Trouble concentrating
**Cognitive Total Number of Items**	**3 items**	**3 items**

**Mental-Emotional Domain**		
**Item #**	**Original HDQ Items**	**Final SF-HDQ Items**
24	Feel anxious	Feel anxious
25	Feel sad, down, or depressed	Feel sad, down, or depressed
26	Afraid for my future	Afraid for my future
27	Lack confidence around others	Lack confidence around others
28	Uncomfortable with how my body looks	--
29	Feel isolated	--
30	Feel embarrassed around others	--
31	Feel guilty	--
32	Feel lonely	Feel lonely
33	Discouraged about future life options	--
34	Feel 'shut out' by friends or family	--
**Mental Total Number of Items**	**11 items**	**5 items**

Item #	Original HDQ Items	Final SF-HDQ Items
**Uncertainty Domain**		
35	Worry about future health living with HIV	Worry about future health living with HIV
36	Worry about lab test results such as my CD4 count and viral load	Worry about lab test results such as my CD4 count and viral load
37	Worry about having a serious illness	--
38	Worry about what the outcome of next episode of illness might be	--
39	Worry about the side effects of HIV treatments	Worry about the side effects of HIV treatments
40	Worry about income or financial security living with HIV	Worry about income or financial security living with HIV
41	Worry what might happen to my family and friends if have an episode of illness	Worry what might happen to my family and friends if have an episode of illness
42	Worry about being able to remain in the workforce or return to the workforce. *(0=not at all or not applicable)*	--
43	Worry about dying	--
44	Worry about my bodily appearance	--
45	Worry about the legal issues of telling others about my HIV status	--
46	Worry about what others would think of me if they knew I was HIV positive	--
47	Worry about transmitting HIV to others	--
48	Put certain life decisions on hold (such as buying a house, returning to work or school, or starting a family) because of my uncertainty living with HIV.	--
**Uncertainty Total Number of Items**	**14 items**	**5 items**
**Notes:** HDQ43 – removed as this item considered to be captured by HDQ35 worry about future health (see Additional file 1).		

**Day-to-Day Activities Domain**		
**Item #**	**Original HDQ Items**	**Final SF-HDQ Items**
49	Unsteady on my feet	Unsteady on my feet
50	Trouble walking	Trouble walking
51	Trouble climbing stair	--
52	Trouble with daily activities such as eating, bathing, grooming, or dressing	--
53	Trouble doing household chores such as cleaning, doing dishes, laundry, and cooking	Trouble doing household chores such as cleaning, doing dishes, laundry, and cooking
54	Trouble taking part in leisure or recreation, such as exercise or dancing	Trouble taking part in leisure or recreation, such as exercise or dancing
55	Trouble getting out to do errands, such as grocery shopping, banking, or doctor's appointments	Trouble getting out to do errands, such as grocery shopping, banking, or doctor's appointments
56	Trouble keeping track of my finances	--
57	Trouble getting around, such as driving or taking public transportation	--
**Day Total Number of Items**	**9 items**	**5 items**
**Notes:** HDQ57 - Deleted because DIF in graph analysis for country and this could be considered part of HDQ55 (trouble getting out to do errands (see Additional File 1).		

**Social Domain**		
**Item #**	**Original HDQ Items**	**Final SF-HDQ Items**
58	Find hard to meet the needs of those I care for	Find hard to meet the needs of those I care for
59	Find it hard to fulfill role as a family or community member living with HIV	--
60	Feel cut off from friends, networks, ethnic or religious communities	--
61	Illness prevents from doing volunteer or paid work or going to school	--
62	Feel work performance limited (0=not at all or not applicable)	Feel work performance limited (0=not at all or not applicable)
63	Struggle to maintain safe and stable housing	Struggle to maintain safe and stable housing *(Rescored to 3 response categories (0-2))*
64	Find it hard to talk with others about illness, even family and friends	--
65	Find it hard to ask others for help when go through an episode of illness	Find it hard to ask others for help when go through an episode of illness
66	Find it hard to start new friendships living with HIV	--
67	Find it hard to start new intimate, sexual relationships living with HIV (0=not at all or not applicable)	Find it hard to start new intimate, sexual relationships living with HIV (0=not at all or not applicable) *(Rescored to 3 response categories (0-2))*
68	Tend to isolate myself from others because I am HIV positive	Tend to isolate myself from others because I am HIV positive
69	Find it hard to take part in leisure or recreational things because can't afford it	Find it hard to take part in leisure or recreational things because can't afford it
**Social Total Number of Items**	**12 items**	**7 items**
**Notes:** Rescoring: Rescored to 3 response categories (0-2) - HDQ63 and HDQ67 to achieve ordered thresholds; HDQ61: Deleted HDQ61 because captured in HDQ62 – which describes limitations rather than prevention (all or nothing).		
