# Supplementary material for: Developing a short-form version of the HIV Disability Questionnaire (SF-HDQ) for use in clinical practice: a Rasch analysis
Source: Health Qual Life Outcomes. 2021 Jan 6;19:6. doi: 10.1186/s12955-020-01643-2 (PMC7789190; doi:10.1186/s12955-020-01643-2)
Supplement: Supplementary file 4 — Additional file 4 Scoring Algorithms for SF-HDQ Domains. [file 12955_2020_1643_MOESM4_ESM.pdf]

Additional File 4: Scoring Algorithms for SF-HDQ Domains
(Total = Raw Domain Sum Score)

Physical Domain (10 items)

$$\text{Scaled Score} = 51.21609 + 13.93883 \text{ (-3.674347 } + 0.435572 \times Total + \text{-0.018692} \times Total^2 + 0.000314 \times Total^3)$$

Cognitive Domain (3 items)

$$\text{Scaled Score} = 48.34017 + 9.369442 \text{ (-5.159343 } + 1.283297 \times Total + \text{-0.112806} \times Total^2 + 0.006665 \times Total^3)$$

Mental-Emotional Domain (5 items)

$$\text{Scaled Score} = 52.04952 + 13.33447 \text{ (-3.903382 } + 0.803233 \times Total + \text{-0.059565} \times Total^2 + 0.001908 \times Total^3)$$

Uncertainty Domain (5 items)

$$\text{Scaled Score} = 49.72724 + 14.0912 \text{ (-3.528956 } + 0.723459 \times Total + \text{-0.055904} \times Total^2 + 0.001874 \times Total^3)$$

Day-to-Day Activities Domain (5 items)

$$\text{Scaled Score} = 44.84344 + 11.43029 \text{ (-3.92321 } + 0.803392 \times Total + \text{-0.068271} \times Total^2 + 0.002499 \times Total^3)$$

Social Domain (7 items)

$$\text{Scaled Score} = 47.1352 + 14.26797 \text{ (-3.303567 } + 0.627115 \times Total + \text{-0.046006} \times Total^2 + 0.001335 \times Total^3)$$
